# Phase I clinical trial of combination imatinib and ipilimumab in patients with advanced malignancies

**DOI:** 10.1186/s40425-017-0238-1

**Published:** 2017-04-18

**Authors:** Matthew J. Reilley, Ann Bailey, Vivek Subbiah, Filip Janku, Aung Naing, Gerald Falchook, Daniel Karp, Sarina Piha-Paul, Apostolia Tsimberidou, Siqing Fu, JoAnn Lim, Stacie Bean, Allison Bass, Sandra Montez, Luis Vence, Padmanee Sharma, James Allison, Funda Meric-Bernstam, David S. Hong

**Affiliations:** 1 Department of Cancer Medicine, The University of Texas MD Anderson Cancer Center, 1515 Holcombe Blvd, Houston, TX 77030 USA; 2 Institute for Personalized Cancer Therapy, The University of Texas MD Anderson Cancer Center, 1515 Holcombe Blvd, Houston, TX 77030 USA; 3 Department of Investigational Cancer Therapeutics, The University of Texas MD Anderson Cancer Center, 1515 Holcombe Blvd, Houston, TX 77030 USA; 4 Sarah Cannon Research Institute at HealthOne, 1800 Williams Street, Suite 300, Denver, CO 80218 USA; 5 Pharamacy Clinical Programs, The University of Texas MD Anderson Cancer Center, 1515 Holcombe Blvd, Houston, TX 77030 USA; 6 Department of Immunology, The University of Texas MD Anderson Cancer Center, 1515 Holcombe Blvd, Houston, TX 77030 USA; 7 Department of Cancer Medicine, John Mendelsohn Faculty Center (FC8.3050), The University of Texas MD Anderson Cancer Center, 1515 Holcombe Blvd., Unit 0455, Houston, TX 77030 USA

## Abstract

**Background:**

Imatinib mesylate can induce rapid tumor regression, increase tumor antigen presentation, and inhibit tumor immunosuppressive mechanisms. CTLA-4 blockade and imatinib synergize in mouse models to reduce tumor volume via intratumoral accumulation of CD8+ T cells. We hypothesized that imatinib combined with ipilimumab would be tolerable and may synergize in patients with advanced cancer.

**Methods:**

Primary objective of the dose-escalation study (3 + 3 design) was to establish the maximum tolerated dose (MTD) and recommended phase II dose. Secondary objectives included evaluation of antitumor activity of the combination based on KIT mutation status and the capacity of tumor-associated immune biomarkers to predict response.

**Results:**

The primary objective to establish the maximum tolerated dose (MTD) was achieved, and the recommended phase II doses are ipilimumab at 3 mg/kg every 3 weeks and imatinib 400 mg twice daily. Of the 35 patients treated in the escalation and GIST expansion, none experienced dose-limiting toxicities. The most common grade 1/2–related adverse events (AEs) were fatigue (66%), nausea (57%), anorexia, vomiting (each 31%), edema (29%), and anemia, diarrhea, and rash (each 23%). Grade 3 AEs occurred in 6 patients (17%) and included fatigue, anemia, fever, rash, and vomiting. There were no grade 4 AEs. In general, the combination was well tolerated. Among all patients, 2 responses were seen: 1 partial response (GIST) and 1 partial response (melanoma). Stable disease was seen in 6 patients lasting an average of 6 months. The melanoma responder was KIT mutated and the GIST responder was wild-type.

**Conclusions:**

Our findings suggest that this combination of a targeted agent with checkpoint blockade is safe across multiple tumor types. Low activity with no clear signal for synergy was observed in escalation or GIST expansion cohorts. Assessment of antitumor activity of this combination in the KIT-mutant melanoma population is being evaluated.

**Trial registration:**

Clinicaltrials.gov NCT01738139, registered 28 November 2012.

**Electronic supplementary material:**

The online version of this article (doi:10.1186/s40425-017-0238-1) contains supplementary material, which is available to authorized users.

## Background

Cytotoxic T-lymphocyte antigen 4 (CTLA4) is expressed on the surface of T-cells and plays a role in suppressing T-cell activation [1]. CTLA-4 blockade using anti-CTLA4 monoclonal antibodies has become a promising therapeutic strategy for promoting anti-tumor immune response. There is now strong evidence that durable antitumor responses can be achieved with anti-CTLA-4 checkpoint blockade therapy. Across multiple studies, approximately 20% of patients with metastatic melanoma who were treated with ipilimumab survive ≥ 5 years [2, 3]. Unfortunately, this success has not been replicated to date across other tumor types. Even in melanoma, there is a need to improve response rates and ultimately increase the number of patients cured. To build upon the success of anti-CTLA-4 blockade, an ideal strategy is to selectively combine ipilimumab with other agents to improve its established efficacy.

Imatinib mesylate is a small-molecule inhibitor of the *KIT* tyrosine kinase, often found in gastrointestinal stromal tumors (GISTs) and melanoma tumors (including mucosal, acral, and those arising from chronically sun damaged skin). Up to ninety-five percent of GIST tumors express the c-KIT receptor [4, 5], and roughly 85% are driven by oncogenic mutations in the KIT gene [6]. Similarly, c-KIT protein expression has been reported in up to 90% of mucosal melanoma tumors and roughly 20% harbor KIT mutations [7, 8]. Imatinib has been shown to induce an 80% clinical response in patients with advanced GISTs and to dramatically increase overall survival [9, 10]. Likewise, imatinib therapy has also shown promising tumor responses in *KIT*-mutant melanomas [11]. Indeed, imatinib has set the standard for the potential of oncologic therapies targeting signaling pathways. In addition to the direct effect on tumor cells, there is mounting evidence that imatinib plays a critical role via indirect modulation of the tumor immune microenvironment. Rusakiewicz et al. [12] describe a T-cell and NK-cell immune-surveillance system in GISTs that is enhanced by imatinib therapy through down-regulation of major histocompatibility complex class I molecules on tumor cells. Tumor infiltration by these immune cells was associated with improved progression-free survival in association with KIT exon 11 mutations. More recent data suggest that tyrosine kinase inhibitors can induce a Th1-specific T-cell response [13] and reduce myeloid-derived suppressor cells, both of which are thought to promote antitumor immune activity [14]. Furthermore, imatinib therapy has been shown to enhance tumor-antigen presenting cell function [15]. Because of its ability to inhibit tumor immunosuppressive mechanisms and increase tumor antigen presentation from direct tumor cell killing, imatinib is a promising candidate to synergistically enhance the antitumor T-cell activation generated by CTLA-4 blockade immunotherapy.

In preclinical models of GISTs, Balachandran et al. [16] convincingly demonstrated the potential benefit of combining imatinib with anti-CTLA-4 checkpoint blockade. They demonstrated that combination therapy in mouse models of GIST synergistically reduces size of tumors mediated by imatinib-dependent intratumoral accumulation of CD8+ T-cells and suppression of Treg-cells. This phase I clinical trial was designed to identify the maximum toxic dose (MTD) of imatinib mesylate plus ipilumumab combination therapy and to test the effectiveness of this combination to treat patients with advanced malignancies.

## Methods

### Patients

Major inclusion criteria were metastatic or unresectable solid tumor refractory to standard therapies, age ≥15 years, ECOG ≤2, and normal organ and bone marrow function. For expansion cohorts, patients were required to have a metastatic or unresectable GIST, melanoma, or uncategorized tumors with tumor biopsies testing positive for *KIT* mutations or c-KIT expression on immunohistochemical analysis. Notably, all expansion cohort patients had *KIT* mutations confirmed by molecular testing prior to inclusion. Major exclusion criteria included active autoimmune disease, human immunodeficiency virus (HIV), hepatitis B, hepatitis C, current immunodeficiency disease, and pregnancy. Concurrent immune or vaccine therapies were not allowed. The trial was conducted at single site and was approved by The University of Texas MD Anderson’s Institutional Review Board. Written informed consent was obtained from all patients and all methods were performed in accordance with the protocol specified guidelines and regulations Additional file 1. The phase I escalation portion of this trial has concluded accrual and was registered with ClinicalTrials.gov on November 28, 2012 as NCT01738139.

### Study design

This study was open-label and utilized a 3 + 3 dose-escalation design. Patients received imatinib mesylate at 400 mg either daily or twice daily and ipilimumab at either 1 mg/kg or 3 mg/kg on day 1 of each 21-day cycle for up to 4 cycles. In the escalation phase, there was a 14-day cycle of imatinib monotherapy preceding cycle 1 to assess for toxicities related to the single agent. If a patient had grade 3 or higher toxicities, the patient was deemed ineligible to start cycle 1 and replaced in the study. All patients with grade 2 or lower toxicities were eligible to proceed into cycle 1 of ipilimumab/imatinib combination therapy and subsequent assessment. Each patient was assessed for dose-limiting toxicities (DLTs) at the end of each cycle. Patients were assessed for response criteria every 2 cycles; clinical decisions were based on response in solid tumor (RECIST) criteria. After 2 cycles, if patients experienced no DLTs, stable disease, partially responsive disease, or nonprogressive disease, they could continue for up to 4 cycles of combination therapy. Patients demonstrating stable disease, partially responsive disease, or nonprogressive disease after cycle 4 could continue daily imatinib mesylate therapy with restaging every 2 cycles (6 weeks) until progression.

The expansion cohort used the MTD determined by the dose escalation study to treat patients with *KIT*-mutant or c-KIT expressing solid tumors. Use of previously obtained tumor biopsy/tissue specimens was acceptable. KIT positive tumors were confirmed by Clinical Laboratory Improvement Amendment (CLIA) certified mutational analysis including isolation of genomic DNA from the tumor; polymerase chain reaction (PCR) amplification of exons 8, 9, 11, 13, and 17 of the c-KIT gene and of regions previously reported to carry gain-of-function KIT mutations (16, 18, 43, 44); bidirectional sequencing of PCR amplicons; and computational analysis of sequences to determine mutation presence or absence. Any tumors harboring mutations found within exons 8, 9, 11, 13, and/or 17 that resulted in any change in amino acid sequence, which included the most commonly found KIT mutations within the juxtamembrane domain of c-KIT (16, 18, 43, 44), were considered positive KIT tumors. Duplications were detected in the patient samples relative to the reference wild-type DNA, but due to the limits of PCR based technique degree of amplification was not quantifiable. Tumor samples that demonstrated strong and diffuse c-KIT expression on immunohistochemical analysis were accepted. These patients were treated with up to 4 cycles of combination therapy at the MTD. In the expansion, both therapeutics were started on day 1; there was no 14-day imatinib lead-in. As in the escalation portion, patients demonstrating stable disease, partially responsive disease, or nonprogressive disease after cycle 4 could continue daily imatinib mesylate therapy until progression.

### Dose-limiting toxicity and maximum tolerated dose

Major criteria for a DLT was grade ≥3 for nonhematologic/laboratory toxicities or ≥4 for hematologic/laboratory toxicities that were at least possibly related to the combination of imatinib and ipilimumab. Other criteria for DLTs included grade 3 fatigue lasting more than 7 days, grade 4 hematologic adverse events (AEs) lasting more than 5 days, and grade 4 neutropenia or thrombocytopenia lasting at least 7 days. All toxicities were assessed via Common Terminology Criteria for Adverse Events (CTCAE) ver. 4.0. For this study, an *immune-related AE* was defined as an AE of unknown etiology, associated with drug exposure, and consistent with an immune phenomenon. The MTD was defined as the highest dose level with less than 2 patients with a DLT from at least 6 patients in the cohort.

### Safety assessments: screening, baseline, and follow-up

Baseline and screening assessment included physician evaluation with history and complete physical examination including prior treatment history and baseline toxicities, and clinical laboratory tests including pregnancy test and urinalysis, electrocardiogram, and baseline imaging. Safety assessments were performed before every cycle of ipilimumab and included monitoring and recording all AEs, documentation of concomitant medications, routine laboratory blood tests, focused history, and a physical examination. After the fourth cycle of ipilimumab, a patient follow-up with toxicity assessment, image evaluation, and routine labs occurred every 1–3 months at the discretion of the treating oncologist. Collection of serum biomarkers was optional and performed under institutional protocol PA13-0291 at baseline and once per cycle. This protocol allows for collection and processing of patient samples by the institutions immunotherapy platform.

### Response

Imaging was obtained at baseline, during the second and fourth cycles of ipilimumab, and at subsequent follow-up visits. Response and progression were evaluated in this study according to guidelines proposed by both RECIST 1.1 and immune-related response criteria (irRC). All measurements were in metric notation. All baseline evaluations were performed within 4 weeks of the beginning of treatment. All clinical decisions were based upon RECIST measurements.

### Peripheral blood mononuclear cell (PBMC) immune correlates

PBMCs were obtained and analyzed via the MD Anderson immunotherapy platform via protocol PA2013-0219. Blood draws were obtained at baseline, before the first dose of ipilimumab, and after each dose of ipilimumab. Lymphocyte counts were obtained from a complete blood count with differential drawn at the same time as the correlative analysis blood draw and analyzed by flow cytometry. After appropriate forward/side scatter and live cells selection, peripheral lymphocytes were gated on CD3 to evaluate CD4/CD8 T-cell surface marker expression. To assess changes in the activation profile among T-cell populations, the following immune markers were stained and a proportion of expression quantified by flow cytometry: 4-1BB, OX40, ICOS, CTLA-4, and PD-1. The absolute lymphocyte count (ALC), absolute monocyte count (AMC), and absolute neutrophil count (ANC) were obtained from the complete blood count with differential and compared between groups of patients based on response.

### Statistical analysis

Demographics, safety, and efficacy data were evaluated via descriptive statistics. Categorical data were summarized as frequency and percentages, whereas continuous variables were summarized as medians and ranges. Differences in PBMCs were assessed with use of the paired Student *t* test. PBMC marker analyses were considered exploratory and results were interpreted with caution due to the high level of Type I errors (due to multiple testing of statistical hypotheses) and Type II errors (due to modest numbers of patients in groups being compared). All p-values were 2-sided. The Kaplan-Meier product limit method was used to estimate overall and progression-free survival distributions. Statistical analyses were performed with use of Excel 2010 (Microsoft Corp.)

## Results

### Baseline patient characteristics

Between March 10, 2013 and January 28, 2015, 50 patients were screened for the dose escalation cohort, and 26 patients were enrolled in the dose escalation phase of this study. Of the 24 patients whom did not enroll, half did not receive insurance approval for ipilimumab; the remaining patients either enrolled on another study or progressed while awaiting approval. While the study was designed as a traditional 3 + 3 escalation a total of 6 patients were enrolled at each dose level due to investigator discretion. Of note, ipilimumab was obtained through off-label insurance approval for this study. Approval was obtained in approximately half of patients screened. Two patients came off study during the 14 day lead in before receiving the first dose of ipilimumab; one at dose level 1 due to intolerance of imatinib and the second at dose level 4 due to rapid disease progression. The remaining 24 patients were evaluated for toxicity and response. The median age was 55 years; 62% of patients were male; and 21 patients had ECOG performance status of 1 or better at the time of enrollment. Most patients had the following types of malignancy: melanoma, 7 patients; renal cell carcinoma, clear-cell type, 5 patients; and non–small cell lung cancer, 3 patients. Patients were in general heavily pretreated, with an average of four prior treatment regimens. Tissue mutation analysis was available for 19 patients. The most common mutations seen in at least 2 patients included KIT in 8 patients, NRAS in 3 patients, TP53 in 3 patients, MET in 2 patients, and PIK3CA in 2 patients [full list available as Additional file 2: Table S1]. Patient characteristics at trial entry are listed in Table 1. Patient treated in the escalation received an average of 3 cycles of ipilimumab.

**Table 1:** Baseline characteristics

	Dose escalation (*n* = 26)	GIST expansion (*n* = 9)
Age	53.3 years (range: 23–71)	61.6 years (range 45–75)
Sex		
Female	10 (38.5%)	2 (22.2%)
Male	16 (61.5%)	7 (77.8%)
ECOG PS		
0	4	0
1	19	9
2	3	0
Malignancy		
Melanoma	8	0
RCC (clear cell)	5	0
NSCLC	3	0
GIST	3	9
Other	7^a^	0
Prior treatment regimens (avg, range)	4 (0–11)	4 (1–6)
Prior anti-CTLA4	1	0
Prior imatinib	3	9
Prior regorafenib^b^	--	8
Prior sunitinib^b^	--	7
Prior nilotinib^b^	--	3
Prior sorafenib^b^	--	1
Molecular profile		
KIT	8	9
Exon 5	1	0
Exon 9	1^c^	0
Exon 10	4	0
Exon 11	2	8^c^
Exon 13	0	2^c^
Exon 17	1^c^	2^c^
NRAS	3	1
TP53	3	0
KDR	2	0
MET	2	0
PIK3CA	2	0
KRAS	1	0

*ECOG PS* Eastern cooperative group performance status, *GIST* gastrointestinal stromal tumor, *RCC* renal cell carcinoma, *NSCLC* non-small cell lung cancer

^a^Prostate, Mesothelioma, Osteosarcoma, Germ-cell tumor, Salivary duct, Renal medullary, Anal

^b^Prior TKI exposure reported for GIST expansion only

^c^Represent patients with co-mutations

A total of 9 patients with *KIT*-mutant GISTs were enrolled between December 1, 2014 and February 29, 2016 in the expansion cohort, and all had previously received at least 2 lines of therapy including imatinib. The median age of these patients was 63 years; 78% were male. Eight patients had known KIT mutations at exon 11 including two with co-mutations in exon 17 and one with a co-mutation in exon 13. One patient had a mutation in exon 13. Patients treated in this expansion arm received an average of 3 cycles of ipilimumab. Accrual to this expansion was discontinued due to slowed enrollment after the lack of responses observed in this population.

### Safety

Treatment-related AEs are listed in Table 2. The most common AEs occurring in at least 10% of subjects were fatigue, nausea, vomiting, anorexia, anemia, edema, diarrhea, rash, shortness of breath, constipation, neuropathy, thrombocytopenia, and infection. Most of these events were mild to moderate in severity and easily managed with supportive treatments. The observed rash was typically maculopapular with associated erythema. Rash occurred during cycles 1 or 2 with similar frequency at both 1 mg/kg and 3 mg/kg ipilimumab doses. Grade 3 or higher toxicities were seen in 6 patients (17%), none of which were dose limiting. Dose-limiting colitis (grade 3–4) was not observed at either dose level of ipilimumab. One patient experienced a grade 3 maculopapular, erythematous rash, nausea, and vomiting, probably related to ipilimumab treatment at the higher dose level of 3 mg/kg. The rash developed during cycle 2, was self-limited, and improved to grade 2 within a few days without intervention. He was hospitalized in cycle 4 for supportive treatment for the nausea and vomiting, which improved with steroids. One patient experience non-neutropenic fevers after cycle 1 and was admitted for monitoring. Ultimately, no infectious source was found for his fevers and he was discharged improved after several days without immunosuppressive medications. Two patients experienced unrelated grade 3 thrombocytopenia after cycle 4 that resulted in treatment delay. Significant AEs included grade 3 hypoglycemia and grade 3 hyperbilirubinemia, both of which were unrelated to study medications. The overall toxicity profile for the 24 patients dosed among the 4 escalation cohorts is summarized in Table 2. No DLTs were encountered, and no treatment-related deaths occurred.

**Table 2:** Treatment Related Adverse Events occurring in > 10% of subjects OR ≥ grade 3

Adverse events	Grade 1–2	Grade 3	Grade 4
Fatigue	23 (65.7%)	1 (2.9%)	
Nausea	20 (57.1%)		
Anorexia	11 (31.4%)		
Vomiting	11	1	
Edema	10 (28.6%)		
Anemia	8 (22.9%)	1	
Diarrhea	8		
Rash	8	1	
Shortness of breath	7 (20%)		
Constipation	5 (14.3%)		
Thrombocytopenia	4 (11.4%)		
Fever		1	
Hypoglycemia		1	

### Clinical activity

**Fig. 1:**
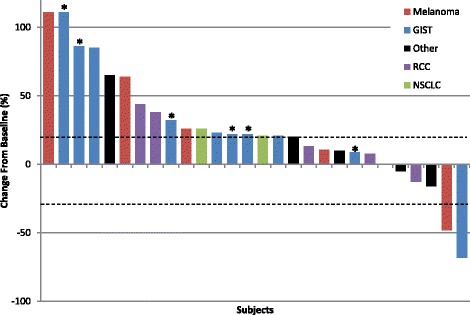
Waterfall plot of maximal responses in evaluable patients (*n* = 28 patients who were imaged at progression, *Expansion GIST patient). [GIST = gastrointestinal stromal tumor; RCC = renal cell carcinoma; NSCLC = non-small cell lung cancer]

Among all patients treated in the escalation phase, 2 responses were observed by RECIST criteria, with an overall response rate of 8.3% (2/24 patients). Among 9 patients with KIT-mutated GISTs who previously progressed on imatinib monotherapy treated at the MTD in the expansion arm, no responses were seen. Figure 1 is a waterfall plot representation of the best response among 28 evaluable patients (3 dose escalation and 2 expansion patients had clinical progression and no available restaging imaging). Both responses were seen at or above dose level 3. The first patient had a wild-type gastric GIST, strongly positive for c-KIT expression on immunohistochemistry, but without detectable *KIT* mutation. She had a history of prior treatment with surgical excision and had received imatinib at 400 mg daily before experiencing metastatic disease in the liver and lungs. She was enrolled at dose level 3, consisting of treatment with imatinib at 400 mg oral daily and intravenous ipilimumab at 3 mg/kg. After 2 cycles of treatment, she had a partial response with a 68% reduction in her disease by RECIST measurement. At the time of data cutoff, June 3rd 2016, her disease remained stable and she had continued to receive imatinib on study for 16 months.

The second patient had a KIT-mutated vulvar melanoma that had been treated surgically before subsequent development of lung metastatic disease. Previous testing had demonstrated a KIT gene missense mutation in codon 576 of exon 11 (CTT to CCT) that would change the amino acid from leucine to proline. She was enrolled at dose level 4 and treated with imatinib at 400 mg orally twice daily and intravenous ipilimumab at 3 mg/kg. After 2 cycles, the patient experienced a 21% decrease in the size of her lung nodules. After 4 cycles, her disease had minimally increased in size. She continued on trial, and restaging after cycles 6 and 8 demonstrated a confirmed partial response to therapy with reductions of 40 and 48% from baseline, respectively. This response was stable through cycle 10 of therapy. During cycle 11, the patient presented with dizziness and was found to have numerous brain metastases; she was taken off trial after nearly 10 months on study.

**Fig. 2:**
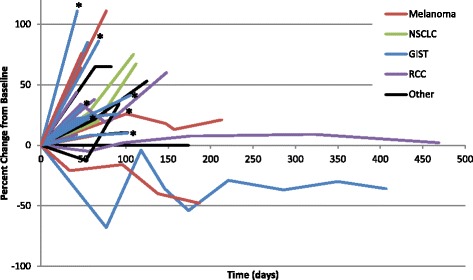
Scatter plot of individual patient responses over time in evaluable patients (*n* = 28, *Expansion GIST patient). [GIST = gastrointestinal stromal tumor; RCC = renal cell carcinoma; NSCLC = non-small cell lung cancer]

A total of 6 additional patients experienced stable disease that lasted at least 12 weeks on therapy. This included 2 patients with melanoma, and 1 each of the following tumor types: anal squamous cell, clear cell renal, prostate, and peritoneal mesothelioma. Notably, none of these patients had a detectable *KIT* mutation. 2 patients developed progressive disease after 12 weeks on study, 3 patients withdrew consent, and one patient with RCC remained on therapy with stable disease for 15 months (Fig. 2).

### Immune cell correlates of response

Peripheral blood samples were collected and processed through our institutional immunotherapy platform. Flow cytometric analysis was performed on samples providing detailed information about the subpopulations of immune cells. Due to timing of protocol activation, a small number of samples were collected from enrolled patients, limiting broader conclusions about the relationship between immune surface marker changes and clinical response. 4-1BB, CTLA4, ICOS, OX40, and PD-1 expression on CD4 and CD8 T cells was measured in 5 patients on PBMCs collected on at least 2 separate time points (Additional file 3). There was a trend toward increased ICOS and OX40 expression on CD4+ T cells after ipilimumab treatment; this was not appreciated on CD8+ T cells. An exploratory analysis of changes in nucleated blood cells was performed on all 24 evaluable patients using results of complete blood counts collected during and off treatment. Specifically, the absolute neutrophil counts (ANC), ALC, and absolute monocyte count (AMC) were evaluated for change from baseline over the course of treatment and correlated with patient response. One notable finding was that the ALC was significantly more likely to decrease over the course of treatment in patients with progressive disease than in those with stable disease (*p* < 0.05), [Additional file 4: Table S2; ALC baseline, mean, and range in Additional file 5: Figure S1]. No other differences were noted in total white blood cell count, ANC, or AMC based on response to therapy [Additional file 4: Table S2].

## Discussion

The primary objectives of this study were achieved and demonstrate that the combination of ipilimumab and imatinib is safe and well tolerated. Since no DLTs were encountered, the MTD was the highest evaluated dose level: intravenous ipilimumab at 3 mg/kg every 3 weeks and imatinib mesylate at 400 mg orally twice daily. Toxicities at these doses were most often grades 1 or 2 and easily managed with supportive treatments. The observed grade 3 toxicities included nausea, rash, cytopenias, and fatigue. These toxicities are all commonly seen with imatinib, but could also be immune-related AEs. These higher-grade toxicities were generally self-limited and not more severe than those seen in ipilimumab monotherapy trials [17, 18]. This is an important finding given the concern for enhanced toxicity with ipilimumab-based combinations. In this study, toxicities of the combination did not appear to be worse than either agent in monotherapy. In future studies, combining correlative studies of immune/inflammatory response with incidence of adverse events may help to better elucidate the mechanisms underlying observed toxicities.

Responses with the combination were seen in one patient with a GIST and one patient with melanoma. Of interest, the responder with a GIST did not have an identifiable mutation in *KIT*, whereas the patient with melanoma had a mutation in exon 11. Wild-type c-KIT expressing GIST and *KIT*-mutated melanoma represent minority subgroups of their respective diseases [19–21] with limited treatment options, highlighting the need for new therapeutic developments. With such a limited number of responses, one should not draw broad conclusions about the efficacy of the combination in these tumor subtypes without further investigation.

The patient with a gastric GIST had been treated with imatinib therapy within 1 year of trial enrollment but was taken off due to disease progression with multifocal metastases in her lungs. While on trial, she experienced a significant reduction in metastatic disease burden, achieving a stable reduction in disease to approximately 40% of baseline. It is impossible to differentiate whether the benefit came from a single agent or the combination. Rapid reduction in tumor burden is more commonly observed with targeted agents than with immunotherapy or chemotherapy [22–24], including with imatinib [25, 26]. However, given her recent prior disease progression while taking imatinib, it seemed unlikely her response would arise from a retrial of the single agent. The phenomenon of pseudoprogression [27] seen with ipilimumab refers to the clinical observation of delayed response after initial progression on therapy. Her pattern of disease control is also inconsistent with delayed response that can be seen with checkpoint blockade. In sum, the patient’s pattern of tumor reduction is more consistent with response to a targeted agent than checkpoint blockade.

The other response was observed in the patient with *KIT*-mutated vulvar melanoma lacking a *BRAF* mutation. Vulvovaginal melanomas are rare, representing less than 1% of all melanomas but 56% of female mucosal melanomas [28]. Among vulvar melanomas, approximately 20% contain *KIT* mutations [29], a number in line with the rate seen among other nonvulvar melanoma subtypes [21]. Limited benefit was seen with imatinib therapy in an unselected population [30], but there has been interest in the use of c-KIT targeting therapies in *KIT*-mutated melanoma. Several trials have reported varying degrees of benefit for the role of tyrosine kinase inhibitors including imatinib [11, 31], nilotinib [32], and dasatinib [33] in *KIT*-mutated melanomas. Notably, this patient had a point mutation in exon 11 at L576P, the most common *KIT* mutation in melanoma [34]. Preclinical data in human-derived melanoma cell lines with the L576P mutation suggest that imatinib in vitro is less effective at inhibiting cell growth than dasatinib due to a lower receptor affinity [35]. However, clinical experience suggests dasatinib may be less effective in targeting this mutation than imatinib [36]. Further, this patient had not been previously exposed to ipilimumab and therefore could be representative of the 11% of patients with advanced melanoma who respond ipilimumab monotherapy [17].

A *KIT*-positive expansion arm enrolled 9 additional patients with *KIT*-mutated GISTs, including patients with mutations at exons 11, 13, and 17. All were negative for *PDGFRA* mutations. Moreover, no patients in this cohort had a mutation at exon 9, likely because such patients were preferentially considered for non-imatinib trials. All 9 patients had been previously treated with imatinib, and all had more recently failed a non-imatinib therapy before study enrollment. Unfortunately, these results suggest that the addition of ipilimumab to imatinib therapy is unlikely to be sufficient to activate an antitumor immune response in heavily pretreated patients with GISTs. A recently published study examining combination of ipilimumab with dasatinib reported limited efficacy with this combination. However, their correlative data suggests that IDO suppression may correlate with antitumor efficacy in GIST [37]. Future investigation should focus on changes to the tumor microenvironment (such as IDO expression) and systemically with therapy and evaluate combination of KIT inhibition with other checkpoint blockade agents.

Of importance, although the side-effect profile of the combination included toxicities observed with each agent in monotherapy, these did not seem exacerbated by the combination, even at higher doses. A critical concern and potential limitation of combination therapies is that cumulative or even synergistic toxicities may lead to an unacceptably high rate of AEs. In our experience, the combination of these agents had a similar toxicity profile compared with either agent in monotherapy. Although the immune profiling of PBMCs was limited, it yields some insights into toxicities of the combination. We observed that patients with progressive disease were significantly more likely to have a decrease in ALC during treatment than were patients with stable disease. Notably, imatinib has been shown to decrease the number of myeloid suppressor cells and improve the responsiveness of T-cells [14]. The decrease in ALC in patients with progression may be representative of a failure to generate an improved systemic T-cell response rather than a suppressive effect of treatment. There is emerging evidence to suggest a high neutrophil-to-lymphocyte ratio is an independent negative prognostic factor in patients with surgically resected GISTs [38]. Although we did not observe any significant response-based differences in this ratio among our patients, this conceptually supports the finding that a higher lymphocyte count may have predictive significance. As described in the preclinical work by Balachandran [16], the mechanism by which imatinib enchanced T cell antitumor activity was via reduced tumor indoleamine 2,3-dioxygenase (Ido) enzyme production. In future immunology trials with imatinib, incorporation of tumor tissue Ido expression and function analysis pre- and post- treatment would provide insight into its relevance in the clinical setting. Further structural and functional studies of circulating lymphocytes in patients treated with similar combination therapies are warranted.

## Conclusions

In summary, the combination of ipilimumab at 3 mg/kg and imatinib at 400 mg orally twice daily is safe and well tolerated. Two responses were seen among 35 patients in a patient with wild-type GIST and in one with *KIT*-mutated melanoma. There were no responses among patients with *KIT*-mutated GISTs, including those in an expansion cohort. This combination merits further investigation in patients with *KIT*-mutated melanoma. The results of an ongoing expansion cohort targeting this population will provide additional evidence as to whether to pursue this combination in *KIT*-mutated melanoma.

## Additional files

Additional file 1: A Phase I Trial of Ipilimumab (Immunotherapy) and Imatinib Mesylate (c-Kit Inhibitor) in Patients with Advanced Solid Malignancies. Description of data: This is a full, up to date copy of the protocol available as supplemental information. (PDF 600 kb)

Additional file 2: Table S1. Mutations in patients with available profiling (*n* = 30). Description of data: A summary of mutations identified in all patients on trial based on available profiling studies. (PDF 19 kb)

Additional file 3: Flow characteristics of patient PBMCs during the course of treatment. Description of data: The raw percentages of CD3+ cells with expression of various surface markers expressed on CD4+ or CD8+ T-cells. (PDF 63 kb)

Additional file 4: Table S2. Average change from baseline among WBC count and subpopulations over the course of treatment. Description of data: A summary of average changes in cell counts over the course of treatment from individual patient baselines. (PDF 67 kb)

Additional file 5: Figure S1. Lymphocyte range during treatment. Description of data: A comparison of average lymphocyte range over the course of treatment between patients with stable disease, progression, or response. Baseline and mean counts during treatment are shown as well as range. (PDF 76 kb)

## Abbreviations

AE: Adverse event
ALC: Absolute lymphocyte count
AMC: Absolute monocyte count
ANC: Absolute neutrophil count
CLIA: Clinical laboratory improvement amendment
CTCAE: Common terminology criteria for adverse events
CTLA-4: Cytotoxic T-lymphocyte antigen 4
DLT: Dose-limiting toxicity
ECOG: Eastern cooperative oncology group
GIST: Gastrointestinal stromal tumor
HIV: Human immunodeficiency virus
irRC: Immune-related response criteria
MTD: Maximum tolerated dose
PBMC: Peripheral blood mononuclear cells
PCR: Polymerase chain reaction
RECIST: Response criteria in solid tumors
