# Supplementary material for: Phase I clinical trial of combination imatinib and ipilimumab in patients with advanced malignancies
Source: J Immunother Cancer. 2017 Apr 18;5:35. doi: 10.1186/s40425-017-0238-1 (PMC5394629; doi:10.1186/s40425-017-0238-1)
Supplement: Supplementary file 1 — A Phase I Trial of Ipilimumab (Immunotherapy) and Imatinib Mesylate (c-Kit Inhibitor) in Patients with Advanced Solid Malignancies. Description of data: This is a full, up to date copy of the protocol available as supplemental information. (PDF 600 kb) [file 40425_2017_238_MOESM1_ESM.pdf]

# A Phase I Trial of Ipilimumab (Immunotherapy) and Imatinib Mesylate (c-Kit Inhibitor) in Patients with Advanced Solid Malignancies

Short Title: Ipilimumab and Imatinib Mesylate in Advanced Solid Tumors

Principal Investigator: David S. Hong, MD
Collaborators: Padmanee Sharma, MD/PhD, James Allison, PhD

Additional Contacts: Ann M. Thomas and Ashley Herrick

Coordinating Center: The University of Texas, MD Anderson Cancer Center

Department: Investigational Cancer Therapeutics

Phone: 713-563-5844

Unit: 455

Protocol Type: Standard Protocol

Protocol Phase: Phase I

# Table of contents

Table of contents ........ 2
1. Treatment Scheme ........ 3
2. Objectives ........ 4
3. Background ........ 4
3.1. Ipilimumab ........ 4
3.2. Imatinib Mesylate ........ 5
3.3. Rationale and Scientific Impact ........ 6
4. ELIGIBILITY CRITERIA ........ 7
4.1. Inclusion Criteria ........ 7
4.2. Exclusion Criteria ........ 8
4.3. Inclusion of Women and Minorities ........ 9
5. Drug Information ........ 9
5.1. Ipilimumab ........ 9
5.2. Imatinib Mesylate ........ 16
6. TREATMENT PLAN ........ 24
6.1. Agent Administration ........ 24
6.1.1. 3+3 Dose Escalation of Ipilimumab and Imatinib Mesylate ........ 24
6.1.2. Expansion Cohort ........ 26
6.2. Dose Limiting Toxicities and Maximum Tolerated Dose ........ 27
6.3. Duration of Therapy and Criteria For Treatment Delay ........ 29
6.4. Immune Related Adverse Events ........ 31
6.5. Infusion Reactions and Fever with Ipilimumab ........ 32
6.6. Treatment of Ipilimumab Related Isolated Drug Fever ........ 33
6.7. Concominant, Prohibited and Restricted Therapies During Study ........ 33
7. Criteria for Evaluation ........ 34
7.1. Evaluations at Baseline ........ 34
7.2. Evaluations During Study ........ 35
7.3. Measurement of Effect ........ 36
7.4. Guidelines for Evaluation of Measurable Disease ........ 36
7.5. Response Criteria ........ 37
7.6. Biomarkers ........ 36
8. Adverse Event Reporting ........ 39
9. Statistical Considerations ........ 41
10. References ........ 44
11. Study Calendars ........ 47
11.1. Dose Escalation Study Calendar ........ 47
11.2. Expansion Cohort Study Calendar ........ 48
12. Appendix ........ 49

## 1. Treatment Scheme

Notice: Treatment schedules shall have a standing window of allowance of +/- 2 days. Any treatment day that falls on a weekend or holiday will be scheduled on the next business day. For treatment or dose modification questions, please contact David Hong, MD by phone (713-563-5844) or e-mail (dshong@mdanderson.org). No investigational or commercial agents or therapies other than those described below may be administered with the intent to treat the patient's malignancy.

**DAY 1-14**: Imatinib mesylate (Gleevec®) will be administered as a daily oral formulation two weeks prior to ipilimumab therapy (14-day run in; before Cycle 1 at dose escalation only) .

**DAY 15-35**: Ipilimumab (Yervoy™) therapy will be given on day 15 as a 90 minute intravenous dose at escalating doses (see escalation schema) in an outpatient setting (Clinical Translational Research Center at MDACC). Daily imatinib mesylate therapy will be continued for three weeks, completing a 21-day treatment cycle.

**Dose Escalation**

| Dose Level | Imatinib | Ipilimumab |
|---|---|---|
| -1 | 400 mg once per day for 35 days | 0.5 mg/kg on day 15 |
| 1 | 400 mg once per day for 35 days | 1 mg/kg on day 15 |
| 2 | 400 mg twice per day for 35days | 1 mg/kg on day 15 |
| 3 | 400 mg once per day for 35days | 3mg/kg on day 15 |
| 4 | 400 mg twice per day for 35 days | 3mg/kg on day 15 |

**Effectiveness of ipilimumab and Imatinib mesylate Combination Therapy: Expansion Cohort**

| Patient Selection | Dose: Ipilimumab/Imatinib Combination Therapy |
|---|---|
| Patients with *KIT* GIST Tumors | MTD |
| Patients with *KIT* confirmed melanoma | MTD |
| Patients with other *KIT* confirmed solid tumors | MTD |

# 2. Objectives

**2.1. Primary Objectives**

a) To evaluate the safety and toxicity profile of intravenous ipilimumab (Yervoy™) administered in combination with oral imatinib mesylate (GLEEVEC®) for patients with advanced malignancies that are refractory to standard therapy, relapsed after standard therapy, or have no available standard therapy.

b) To determine the Maximum Tolerated Dose (MTD) and Dose Limiting Toxicities (DLT) of ipilimumab and imatinib mesylate combination therapy.

**2.2. Secondary Objectives**

a) To determine antitumor activity of ipilimumab and imatinib mesylate combination therapy.

b) To determine antitumor activity of ipilimumab and imatinib mesylate combination therapy in *KIT* confirmed solid tumors.

c) To evaluate the potential predictive role of tumor-associated immune biomarkers for therapy effectiveness.

d) To evaluate the potential predictive role of therapy associated toxicities with antitumor activity.

# 3. Background

Clinical results demonstrate how immunotherapy with ipilimumab can induce long-lasting anti-tumor effects through the generation of anti-tumor immune memory (review clinical trial results (1). However, ipiimumab treatment alone affected only a small proportion of patients. Imatinib mesylate is a small molecule inhibitor of *KIT* tyrosine kinase often found in gastrointestinal stromal tumors (GIST) and mucosal melanoma tumors. Imatinib has shown to induce an 80% clinical response in patients with advanced GIST and dramatically increases overall survival (2, 3). More recently, Imatinib has been shown to selectively reduce GIST tumor immunosuppressive mechanisms (4) suggesting that this particular targeted therapy can enhance anti-tumor immune response. It is suggested that because imatinib mesylate can induce rapid tumor regression, inhibit tumor immunosuppressive mechanisms, and release large amounts of antigenic debris from tumor cell death leading to increased tumor antigen presentation, it may synergistically enhance anti-tumor T-cell activation generated by ipilimumab immunotherapy.

**3.1. Ipilimumab**

T cell activation is a complex process that is initiated when an antigen is presented to the T cell receptor (TCR) followed by the interaction of additional T cell surface molecules with their respective ligands on the antigen presenting cell (APC). This second interaction can result in a positive or a negative costimulatory signal depending on which specific molecules are involved. CTLA-4 is a T-cell surface molecule that, on interaction with the B7 molecule of the APC, leads to the termination of the T-cell response. Blockage of CTLA-4 with the monoclonal antibody Ipilimumab has led to a remarkable enhancement of the immune response in experimental models of cancer and infection (5).

Yervoy™ (ipilimumab) monotherapy has been approved for use in the US (March, 2011), the EU (July, 2011) and Australia (July,2011) for the treatment of patients with unresectable advanced melanoma. Thousands of subjects with several cancer types in 90 completed and ongoing studies, have been treated during its clinical development program, which is focused in melanoma, prostate cancer, lung cancer, and renal cell carcinoma. In melanoma, two Phase 3 studies (**MDX010-20** comparing ipilimumab 3mg/kg to a melanoma-specific vaccine gp100 in pretreated advanced melanoma (6) and **CA184024**, comparing ipilimumab 10mg/kg plus dacarbazine to dacarbazine alone in previously untreated advanced melanoma (7) have demonstrated a survival benefit in patients treated with ipilimumab.

Over half of the patients treated with ipilimumab reported *immune-related adverse events* (irAE) which are defined as any AE associated with drug exposure and consistent with an immune-mediated event (thought to be a consequence of the intrinsic biological activity of ipilimumab). IrAEs predominantly involve the GI tract (manifested most often as diarrhea or colitis) and skin (pruritus and rash), and less commonly the liver (transaminase elevations), endocrine glands (manifested most often as hypophysitis/hypopituitarism) and nervous system (motor neuropathy with or without sensory neuropathy). According to the Investigator's Brochure, most of these irAEs were clinically manageable and reversible with supportive care or corticosteroids.

Efficacy data from Phase I and II studies in melanoma suggest a trend of increasing durability and progression free survival (PFS) rates with increasing doses and duration of exposure to ipilimumab. However, preliminary data suggests that 10mg/kg of ipilimumab is associated with a higher frequency of SAEs and serious (Grade 3 or higher) irAEs than 3mg/kg of ipilimumab (6, 7). It appears that an increased awareness and better management of these side effects has led to a decrease in their severity and an improvement in their control in recent trials. We propose to use the 1-3mg/kg dose administered as 4 doses every 3 weeks and will implement a tight safety rule (see section 5.3) to stop the trial in case of excess toxicity.

It is important to note that because ipilimumab works indirectly through stimulation of the immune system by enhancing T-cell activation, its effect on tumor burden may take weeks to months to become apparent. The clinical activity of ipilimumab may manifest, not only as an early objective response, but also as stable disease (SD) with slow, continuous decline of tumor burden toward response and, in some cases, as a late objective response after initial tumor volume increase. For example, in one study (MDX010-19) the time to first response ranged from day 40 to day 441. Durable responses and SD after treatment with ipilimumab have been observed in several malignancies, including melanoma, prostate and renal cell carcinoma. {Investigator's Brochure, v.10}

### 3.2. Imatinib meslylate

In a pioneering work, Druker and co-workers demonstrated that imatinib mesylate suppressed proliferation of BCR-Abl-positive CML cells in vitro (8). Normal hematopoietic progenitors were largely unaffected (8). It was further discovered that this compound was an effective inhibitor of the PDGF receptor and KIT (CD 117, stem cell factor receptor) tyrosine kinases (9). Imatinib mesylate is specific with 50% inhibiting concentrations (IC50s) of 188nM for c-Abl, 413nM for KIT, 386nM for PDGFR-β. In contrast the IC50s of most of the other cellular tyrosine kinases (8, 10, 11) was found to be > 10 micromolar. These observations laid the groundwork for use of imatinib mesylate in the clinical setting, with potential for killing tumor cells harboring the target kinases without harm to normal host

tissue. The antitumor effects of imatinib mesylate in GIST with activating kit mutations is remarkable (2, 3, 12-14).

Oncogenic *KIT* mutations have been reported in 75% to 80% gastrointestinal stromal tumors (GIST; (15, 16)), testicular seminomas (17), 21 % mucosal melanomas (18, 19), core-binding factor acute myeloid leukemia (CBF-AML; (20)), and systemic mastocytosis (21). Imatinib mesylate has shown to be a good therapeutic option for patients with these different tumor types containing oncogenic *KIT* mutations. Most mutations affect the juxtamembrane region of the *KIT* protein resulting in enhanced downstream signaling of *KIT* and increased cellular proliferation and survival, and can predict responsiveness to imatinib mesylate (22).

GIST is the most common mesenchymal tumor of the gastrointestinal tract. Over 85% of GISTs express the *KIT* receptor (stem cell factor receptor, CD117), as shown by immunohistochemical analysis (23). Approximately 60% of GISTs occur in the stomach, 25% in the small intestine and 10% in the colon and rectum. The remainder arises from other sites in the GI tract or other rare locations such as the gall bladder, appendix, omentum, or mesentery. However, GISTs account for approximately 2% of all stomach tumors, 14% of all small intestine tumors, and 0.1% of colon tumors. The annual incidence is approximately 32,000 new cases a year in the United States (24). The median age at diagnosis is around 58 years (23). As early as the 1940’s, GISTs were often diagnosed as smooth muscle tumors of the GI tract (GI leiomyosarcoma, leiomyoblastoma, and leiomyoma), but advances in histopathology later provided evidence that GISTs were distinct from the smooth muscle tumors.

### 3.3. Rationale and Scientific Impact

One of the suggested limitations of ipilimumab and other immunotherapies is the relatively small proportion of patients who achieve lasting clinical responses. This is likely a result of tumor initiated immunosuppressive responses (25). These immunosuppressive responses can be activation of T-cell CTLA-4 receptors, which suppress T-cell activity, or activation of T-regulatory (T-reg) cell mediated-immunosuppression. Recent pre-clincial studies have demonstrated the potential of enhancing anti-tumor effects of immunotherapy by co-administering targeted therapies. Balachandran et al showed how Imatinib mesylate, a potent inhibitor of *KIT* tyrosine kinase, can synergistically enhance the antitumor activity of CTLA-4 blockade in mouse GIST models by decreasing tumor-associated immunosuppression and enhancing T-cell activity (4). Specifically, imatinib mesylate treatment could directly suppress T-reg activity as well as enhance T-cell localization to tumor sites. This, together with CLT-4 blockade, resulted in dramatic reduction in GIST tumor burden in mice. Furthermore, this study revealed a significant role of T-cells in mediating imatinib anti-tumor activity, further implying a potential combinatorial effect of immunotherapy and targeted therapies. Therefore, it is expected that imatinib mesylate will also enhance anti-tumor effects of Ipilimumab and improve overall patient outcomes in *KIT* positive GIST, melanoma, and other solid tumors.

While Ipilimumab showed dramatic clinical effects in patients with metastatic melanoma, in which there existed no other standard therapy, the response rate, while statistically higher than standard therapy, of ipilimumab monotherapy at the FDA approved dose is still relatively low at 4.2% (26). Furthermore, while imatinib mesylate shows an 80% response rate in patients with GIST, the median progression-free survival of refractory metastatic

GIST patients is < 2-years (27). Balachandran *et al* demonstrated that CTLA-4 blockade and imatinib mesylate combination therapy in mouse models of GIST synergistically reduces size of tumors mediated by imatinib-dependent intratumoral accumulation of $CD8^+$ T-cells and suppression of $T_{reg}$-cells (4). Furthermore, a recent study demonstrated how interferon and imatinib mesylate combination therapy, in a small number of patients, may increase progression-free and overall survival than imatinib mesylate alone (28), demonstrating the high probability of improving clinical outcomes by combining immune- and targeted-therapies for the treatment of cancer. In light of these pre-clinical and clinical studies, we feel confident that our study, in which we combine CTLA-4 blockade (ipilimumab) and imatinib mesylate, will result in increased anti-tumors responses, decrease in tumor size, and potentially increase patients' progression-free survival.

This phase I clinical trial is designed to test the effectiveness of using ipilimumab and imatinib mesylate combination therapy to treat patients with advanced malignancies, which include patients with cancers that are refractory to standard therapy, relapsed after standard therapy, or have no available standard therapy. First, a dose escalation scheme will be implemented in order to identify maximum toxic dose (MTD) of ipilimumab/imatinib mesylate combination therapy. Patients with advanced malignancies will be treated with increasing doses (administered daily) of imatinib mesylate for two weeks prior to escalating doses of ipilimumab and imatinib mesylate combination therapy. Once MTD is determined for combination therapy, this dose will be administered in three treatment cohorts: patients with *KIT* confirmed 1) GIST tumors, 2) melanoma, or 3) uncategorized solid tumors.

Our hypothesis is that: 1) imatinib mesylate and ipilimumab combination therapy will increase anti-tumor response in malignant solid tumors than reported ipilimumab or imatinib mesylate alone studies, via synergistically increasing anti-tumor T-cell activity.

# 4. Eligibility Criteria

To be eligible for this trial, patients must meet all of the following criteria.

### 4.1. Inclusion Criteria

1. For dose escalation study, patients must have histological confirmation of solid tumors that is metastatic or unresectable. For expansion cohorts, patients must have metastatic or unresectable GIST, melanoma, or uncategorized tumors with tumor biopsies testing positive for *KIT* mutations or C-KIT expression in tumor biopsies by immunohistochemistry (see section 6.1.2).
2. Patients who have completed previous therapies 5 drug half-lives or 4-weeks prior to enrollment on study (radiation therapy wash out period will be 2-weeks). This includes an exception of patients with metastatic GIST tumors who are taking maintenance imatinib mesylate therapy. These patients are allowed to remain on imatinib mesylate therapy up to enrollment in this study.
3. Age ≥ 15 years
4. ECOG performance status $\leq$2 (Karnofsky >60%).
5. Patients must have normal organ and marrow function as defined below:
   - leukocytes >3,000/mcL

- absolute neutrophil count >1,500/mcL
- platelets >100,000/mcL
- total bilirubin ≤ 2.0 mg/dL. (Does NOT apply to patients with Gilbert's Syndrome)
- AST(SGOT)/ALT(SGPT) <2.5 X institutional upper limit of normal (patients with liver involvement will be allowed ≤5.0 X institutional upper normal limit) serum creatinine <2.0 mg/dL

6. Patients MUST have recovered from all treatment related toxicities to Grade 1 NCI CTC (v 4.0) in severity.
7. Patients must be willing and able to review, understand, and provide written consent before starting therapy.
8. Patients with histologically proven intracranial glioblastoma, gliosarcoma or anaplastic astrocytoma will be eligible. Patients must have shown unequivocal radiographic evidence for tumor progression by MRI scan as defined by Section 11.6. A scan should be performed within 14 days prior to registration and on a steroid dose that has been stable for at least 5 days. If the steroid dose is increased between the date of imaging and registration, a new baseline MRI is required.
9. Patients in the expansion cohort must also agree to participate in the immunotherapy platform protocol (PA13-0291).

### 4.2. Exclusion Criteria

1. Autoimmune disease: Patients with a history of inflammatory bowel disease (including Crohn's disease and ulcerative colitis) and autoimmune disorders such as rheumatoid arthritis, systemic progressive sclerosis [scleroderma], Systemic Lupus Erythematosus or autoimmune vasculitis [e.g., Wegener's Granulomatosis] are excluded from this study.
2. History of acute diverticulitis, intra-abdominal abscess, GI obstruction, abdominal carcinomatosis or other known risk factors for bowel perforation.
3. Any underlying medical or psychiatric condition, which in the opinion of the Investigator, will make the administration of study drug hazardous or obscure the interpretation of AEs: e.g. a condition associated with frequent diarrhea or chronic skin conditions, recent surgery or colonic biopsy from which the patient has not recovered, or partial endocrine organ deficiencies.
4. Uncontrolled intercurrent illness including, but not limited to, ongoing or active infection, history of congestive heart failure, unstable angina pectoris, cardiac arrhythmia, or psychiatric illness/social situations that would limit compliance with study requirements.
5. Known HIV, Hepatitis B, or Hepatitis C.
6. Any non-oncology vaccine therapy used for prevention of infectious diseases (for up to one month prior to or after any dose of ipilimumab).
7. Concomitant therapy with any of the following: IL-2, interferon or other non-study immunotherapy regimens; cytotoxic chemotherapy; immunosuppressive agents; other investigational therapies; or chronic use of systemic corticosteroids (when used in the management of cancers other than intracranial glioblastoma, gliosarcoma or anaplastic astrocytoma, or when used to treat non-cancer-related illnesses).

8. Patients who do not agree to practice appropriate birth control methods while on therapy.
9. Pregnant women are excluded from this study. Women of child-bearing potential and men must agree to use contraception prior to study entry and for the duration of study participation. Should a woman become pregnant or suspect she is pregnant while participating in this study, she should inform her treating physician.

### 4.3. Inclusion of Women and Minorities

Both men and women of all races and ethnic groups are eligible for this trial. The patient population screened for this study adheres to the referral patterns reported at M.D. Anderson Cancer Center.

# 5. Drug Information

### 5.1. Ipilimumab (Yervoy™): manufactured by Bristol-Myers Squibb Co (BMY).

5.1.1. *Physical/Chemical Properties:*
Ipilimumab is an IgG1 monoclonal antibody. It is a soluble protein consisting of 4 polypeptide chains, 2 identical heavy chains consisting of 467 amino acids and 2 identical light chains consisting of 235 amino acids. It has a projected relative mass (Mr) of 145,424 dalton (d) based on the amino acid sequence.

5.1.2. *Mechanism of Action:*
Ipilimumab is a human immunoglobulin G (IgG1)κ anti-CTLA-4 monoclonal antibody (mAb) that binds to CTLA-4 antigen expressed on the plasma membrane of T cells and blocks the interaction of CTLA-4 with its natural ligands, B7.1 (CD80) and B7.2 (CD86). *In vitro* studies were performed with ipilimumab to demonstrate that it is specific for CTLA-4, actively inhibits CTLA-4 interactions with B7.1 and B7.2, and does not show any binding to human B7.1, B7.2 negative cell lines, demonstrating by immunohistochemistry that ipilimumab is specific and non-cross reactive in non-human primate tissues.

5.1.3. *Pharmacology:*
Ipilimumab is a human immunoglobulin G (IgG1)κ anti-CTLA-4 monoclonal antibody (mAb). In vitro studies were performed with ipilimumab to demonstrate that it is specific for CTLA-4, actively inhibits CTLA-4 interactions with B7.1 and B7.2, does not show any cross-reactivity with human B7.1, B7.2 negative cell lines, and stains the appropriate cells without non-specific cross-reactivity in normal human tissues, as demonstrated by immunohistochemistry. Ipilimumab does cross-react with CTLA-4 in non-human primates including cynomolgus monkeys.

Ipilimumab was originally produced and purified from a hybridoma clone. Subsequently, a transfectoma (CHO cell) has been generated that is capable of producing more ipilimumab on a per cell basis than the hybridoma. Material from the transfectoma will be utilized in this and future ipilimumab clinical studies. Biochemical, immunologic and in vivo preclinical primate assessments demonstrated similarity between hybridoma and transfectoma-derived ipilimumab.

*5.1.4. Pre-clinical Toxicology*

Complete information on the pre-clinical toxicology studies can be found in the ipilimumab Investigator Brochure (IB). Non-clinical toxicity assessments included in-vitro evaluation for the potential of ipilimumab to mediate complement-dependent cellular cytotoxicity (CDCC) or antibody-dependent cellular cytotoxicity (ADCC), and toxicology assessments in cynomolgus monkeys alone and in the presence of vaccines.

The *in vitro* studies demonstrated that ipilimumab did not mediate CDCC of PHA- or (CD)3-activated human T cells. However, low to moderate ADCC activity was noted at concentrations up to 50 ug/mL. These data are consistent with the requirement of high levels of antigen expression on the surface of target cells for efficient ADCC or CDCC. Since ipilimumab is a human IgG1, an isotype generally capable of mediating CDCC and ADCC, the lack of these activities is likely due to a very low expression of CTLA-4 on activated T cells. Therefore, these data suggest that ipilimumab treatment would not result in depletion of activated T cells in- vivo. Indeed, no depletion of T cells or T cell subsets were noted in toxicology studies in cynomolgus monkeys.

No mortality or signs of toxicity were observed in three independent 14-day intravenous toxicology studies in cynomolgus monkeys at multiple doses up to 30 mg/kg/dose. Furthermore, ipilimumab was evaluated in sub chronic and chronic toxicology studies in cynomolgus monkeys with and without Hepatitis B (HepB)Vaccine and Melanoma Vaccine. Ipilimumab was well tolerated alone or in combination in all studies. There were no significant changes in clinical signs, body weight values, clinical pathology values or T cell activation markers. In addition, there were no significant histopathology changes in the stomach or colon.

*5.1.5. Pharmacokinetics of Ipilimumab in Patients*

Pharmacokinetic (PK) profiles for ipilimumab have been analyzed. The primary objective of Protocol MDX-010-015 was to determine the safety and PK profile of single and multiple doses of ipilimumab derived from a transfectoma or hybridoma cell line. This study is still ongoing and data is preliminary. Mean plasma concentrations of ipilimumab administered at dosages of 2.8 mg/kg (transfectoma-derived drug product), 3 mg/kg (hybridoma-derived drug product,), 5 mg/kg and 7.5 mg/kg (transfectoma) appear to be dose-proportional over time. Preliminary PK analyses reveal that the volume variables were approximately that of plasma volume (range of mean apparent volume of distribution at steady state [Vss] across cohorts 2.8, 3, 5, 7.5, 10, 15 and 20 mg/kg was 57.3 to 82.6 mL/kg), indicating drug distribution was mostly limited to the intravascular space. The clearance (Cl) was ,low (range 0.11 to 0.29 mL/h/kg) and reflective of the half-life (range 297 to 414 h). Mean residence time (MRT) was long (range 435 to 538 h), consistent with the long terminal disposition phase of ipilimumab. In general, there was moderate variability in the PK parameters among patients, with coefficient of variation (CV) of 11% to 48% in AUC (0-21d), 20% to 59% in Cl and 17% to 46% in ss. Future clinical studies, including this study, will utilize the transfectoma derived product.

*5.1.6. Clinical Safety*

The safety profile of ipilimumab has been consistent across trials with a) the majority of adverse events being inflammatory in nature and consistent with the proposed mechanism of action of ipilimumab (immune-related adverse events, IRAEs), b) the same types of such immune-mediated events in the GI tract, skin, liver and endocrine system being reported and c) most of these events being manageable with immune

suppressive therapies. Overall, nearly all subjects in clinical studies with ipilimumab reported AEs of any grade and most reported at least 1 AE that was considered treatment related.

1. *Details of Drug-Related Aes and SAEs:*
   Drug-related adverse events (Aes) have been reported in studies with ipilimumab as monotherapy as well as in combination studies with vaccines, cytokines or chemotherapy.

   The AE profile of ipilimumab is relatively well characterized with drug-related Aes mostly being immune-related adverse events (IRAEs), which are considered to be associated with the mechanism of action of ipilimumab. The most common IRAEs are colitis and diarrhea, rash, pruritus, deficiencies of endocrine organs (pituitary, adrenal or thyroid), hepatitis, or uveitis. Rare complications are bowel perforations (~1%) resulting from underlying severe colitis, which have required surgical intervention.

   Drug-related Grade 3 or 4 serious Aes (SAEs) consist mostly of immune-related SAEs and include: rash/desquamation, pruritus, uveitis, speech impairment, abdominal pain, diarrhea/colitis, nausea/vomiting, transaminase elevation, adrenal insufficiency, panhypopituitarism and atrial fibrillation. Please refer to the most recent version of Investigator's Brochure (IB) for the latest update on SAEs.

5.1.7. *Immune-Related Adverse Events (IRAEs):*
Many of the adverse events considered related to ipilimumab appear to be immune in nature and presumably a consequence of the intrinsic biological activity of ipilimumab. An immune-related adverse event (IRAE) is defined as any adverse event associated with drug exposure and consistent with an immune-mediated event. Disease progression, infections and other etiologic causes are ruled out or deemed unlikely as contributing to the event. Supportive data, such as autoimmune serology tests or biopsies, are helpful but not necessary to deem an event an IRAE. Events of unclear etiology which were plausibly "immune mediated" have been conservatively categorized as IRAEs even if serologic or histopathology data are absent. These IRAEs likely reflect a loss of tolerance to some self antigens or an unchecked immune response to gut or skin flora. Some breakthrough of immunity may be inseparably linked to the clinical antitumor activity of ipilimumab.

Pooled analysis of Phase II and Phase III trials of ipilimumab in patients with advanced melanoma showed the following: Of 622 subjects treated with 3mg/kg ipilimumab, 56.8-61.3% reported any IRAEs, 6.3-13% reported Grade 3/4 IRAEs and 0.8-1.1% reported Grade 5 IRAEs. Of 325 patients treated with 10mg/kg ipilimumab, 84.3% reported any drug related AE, and 30.5% reported Grade 3/4 IRAEs (IB v14).

1. **Immune-related gastrointestinal events:** GI IRAEs occurred in 28.2-31.1% (Grade3/4 4.5-7.6%, Grade 5 0-0.9%) of subjects treated with 3mg/kg of ipilimumab and in 36.3% (Grade 3/4: 11.7%; Grade 5: 0%) of subjects treated with 10mg/kg of ipilimumab. The clinical presentation of GI IRAEs included diarrhea, increase in the frequency of bowel movements, abdominal pain or hematochezia, with or without fever. Among approximately 10,000 subjects in the BMS internal safety database, 0.5% (51/10,000) reported colitis that was unresponsive to medical management and necessitated colectomy, or had bowel wall perforations associated with ipilimumab-induced colitis. Fourteen of the 51 subjects died of bowel wall perforation complications. Patients should be carefully monitored for GI symptoms that may be

indicative of immune-related colitis, diarrhea or GI perforation. Diarrhea or colitis occurring after initiation of ipilimumab therapy should be evaluated to exclude infectious or alternate etiologies. In clinical trials, immune-related colitis was associated with evidence of mucosal inflammation, with or without ulcerations, and lymphocytic infiltration. GI IRAEs should be monitored until resolution.

2. **Inflammatory hepatotoxicities**: Hepatic IRAEs were reported in 2.1-3.8% (Grade 3/4: 0-2.3%; Grade 5: 0-0.8%) subjects treated with 3mg/kg of ipilimumab and in 8% (Grade 3/4: 6.8%, Grade 5: 0%) of patients treated with 10mg/kg of ipilimumab. Hepatic IRAEs were mostly clinically silent and manifested as transaminase or bilirubin laboratory abnormalities. Fatal hepatic failure has been reported in clinical trials of ipilimumab. Serum transaminase and bilirubin levels must be evaluated before each dose of ipilimumab as early laboratory changes may be indicative of emerging immune-related hepatitis. Evaluations to exclude other causes of hepatic injury, such as infections, disease progression or medications should be undertaken, Liver function abnormalities should be monitored until resolution. Liver biopsies from subjects who had IR hepatotoxicity showed evidence of acute inflammation (neutrophils, lymphocytes and macrophages).

3. **Endocrine toxicities**: Endocrine IRAEs were reported in 3.4-7.6% (Grade 3/4: 0.9-3.8%, Grade 5: 0%) of subjects receiving 3mg/kg of ipilimumab and in 6.2% (Grade 3/4: 2.5%; Grade 5: 0%) of subjects receiving 10mg/kg of ipilimumab. Ipilimumab can cause inflammation of the endocrine system organs, specifically hypophysitis, hypopitutarism, and adrenal insufficiency and patients may present with nonspecific symptoms, which may resemble other causes such as brain metastasis or underlying disease. The most common presentation includes headache and fatigue. Symptoms may also include visual field defects, behavioral changes, electrolyte disturbances and hypotension. Adrenal crisis as a cause of the patient’s symptoms should be excluded. Based on the available data with known outcome, most of the subjects symptomatically improved with hormone replacement therapy. It is possible that long-term hormone replacement therapy will be required for subjects developing hypophysitis/hypopituitarism after treatment with ipilimumab.

4. **Dermatologic toxicities**: Skin IRAEs were reported in 38.9-42.3% (Grade 3/4 0.8-2.4%, Grade 5 0) of subjects receiving 3mg/kg of ipilimumab and in 51.4% (Grade 3/4: 2.5%; Grade 5:0%) of subjects receiving 10mg/kg of ipilimumab. Skin IRAEs presented mostly as a rash and/or pruritus. Some subjects reported vitiligo associated with ipilimumab administration. Fatal toxic epidermal necrolysis has been reported in clinical trials of ipilimumab.

5. **Neurological toxicities**: Neurological IRAEs were reported in 0-0.5% (Grade 3/4: 0-0.3%, Grade 5: 0-0.3%) of subjects receiving 3mg/kg of ipilimumab and in 0.3%

(Grade 3/4: 0%; Grade 5: 0%) of subjects receiving 10mg/kg of ipilimumab. Neurological manifestations included muscle weakness and sensory neuropathy. Among approximately 10,000 subjects treated in the ipilimumab program as of 24-Jun-2011, 11 (0.1%) cases of Guillain-Barre syndrome and 5 (0.05%) cases of myasthenia gravis considered related to study drug were reported, and 2 of the Guillain-Barre syndromes had a fatal outcome. Patients may present with muscle weakness. Sensory neuropathy may also occur. Unexplained motor neuropathy, muscle weakness, or sensory neuropathy lasting more than 4 days should be evaluated and non-inflammatory causes such as disease progression, infections, metabolic syndromes and medications should be excluded.

6. **Other toxicities**: Other IRAEs were reported in 2.3-3.8% (Grade 3/4: 0.8-1.5%, Grade 5: 0-0.3%) of subjects receiving 3mg/kg of ipilimumab and in 5.2% (Grade 3/4: 2.2%; Grade 5: 0.6%) of subjects receiving 10mg/kg of ipilimumab. Ocular inflammation, manifested as Grade 2 or Grade 3 epislcleritis or uveitis, was associated with concomitant diarrhea in a few subjects (<1%) and occasionally occurred in the absence of clinically apparent GI symptoms. Other presumed IRAEs reported include, but were not limited to, arthiritis/arthralgias, pneumonitis, pancreatitits, autoimmune (aseptic) meningitis, autoimmune nephritis, pure red cell aplasia, noninfective myocarditis, polymyositis, and myasthenia gravis, of which were individually reported for <1% of subjects.

5.1.8. *Onset and Resolution of IRAEs*:
The median time to onset of Grade 3-5 IRAEs in MDX010-20 and Phase 2 studies was 7 to 9 weeks. The time to onset of IRAEs was comparable between the 3- and 10-mg/kg doses. With the recommended treatment guidelines, the median times to resolution of Grades 3-4 IRAEs was 4 to 8 weeks. The time to resolution of IRAEs was comparable between the 3 and 10-mg/kg doses.

*5.1.9. Ipilimumab Dose-dependent Safety Profile*
In MDX010-20, immune-related adverse events (irAEs) occurred in 60% of subjects treated with ipilimumab (**3mg/kg**) and ≥ Grade 3 events occurred in 12-16%. Treatment related Aes leading to discontinuation of therapy were reported in 9.9% of the ipilimumab monotherapy arm vs 3.0% of the gp100 monotherapy arm. The most common (>1%) treatment related Aes leading to discontinuation in the ipilimumab monotherapy arm were colitis (2.3%), diarrhea (1.5%) and uveitis (1.5%). In a pooled 3mg/kg group from the Phase 2 studies, 8.1% of subjects reported treatment-related Aes leading to discontinuation. The most common were hypopituitarism (2.7%), colitis (1.8%) and decreased appetite (1.8%). In MDX010-20, treatment-related deaths (defined as a treatment-related AE with an outcome of death, reported at any time during the study) were reported in 4 subjects (3.1%) in the ipilimumab monotherapy group, 8 subjects (2.1%) in the ipilimumab plus gp100 group and 2 (1.5%) subjects in the gp100 monotherapy group.

In CA184024, any irAEs occurred in 76% of subjects treated with ipilimumab (**10mg/kg**) + DTIC, Grade 3 events were reported in 31.6% and Grade 4 events in 10.1%. Treatment-related SAEs were reported for 47% and 6.8% in the ipilimumab

plus DTIC and DTIC monotherapy groups respectively. The most common events in the ipilimumab plus DTIC group were increased ALT and AST (19% each). Other SAEs reported in ≥5% of subjects in the ipilimumab plus DTIC group included diarrhea (6.5%) and pyrexia (5.7%). There were no treatment-related Aes with an outcome of death in the ipilimumab plus DTIC group and 1 (0.4%) in the DTIC group (GI hemorrhage). In a pooled analysis of 325 patients receiving 10mg/kg ipilimumab therapy, Aes (any grade) were reported in 96.9%, Grade 3-4 Aes in 37.5%, related Aes in 84.3%, Grade 3-4 related Aes in 30.5%, SAEs in 51.7%, related SAEs in 29.2%.

In **CA184022**, 3 dose levels of ipilimumab were studied, including 0.3 (n=72) vs 3 (n=71) vs 10mg/kg (n=71). Overall irAEs were reported in 64.8% and 70.4% of patients treated at 3mg/kg and 10mg/kg respectively, Grade 3-4 irAEs were reported in 7% and 25.4% respectively, GI irAEs in 32.4% and 39.4% respectively, Grade 3-4 GI irAEs in 2.8% and 15.5% respectively, hepatic Grade 3-4 irAEs in 0% and 2.8% respectively, endocrine Grade 3-4 irAEs in 2.8% and 1.4% respectively and skin Grade 3-4 irAEs in 1.4% and 4.2% respectively.

In summary, the safety profile of ipilimumab 10mg/kg remains consistent with the low-dose safety profile in that most of the treatment-related SAEs are characteristic of immune-related toxicity, and most of the IRAEs are reported in the GI, hepatic and endocrine systems. However, the frequency of IRAEs, particularly of high grade events, is higher with 10mg/kg of ipilimumab at multiple doses compared with the IRAE frequency reported for lower doses.

*5.1.10. Drug Related Deaths:*

Based on the data available in the BMS internal safety database as of 24-June-2011, study-drug related deaths based on the investigator’s assessment were reported in 82 subjects. Therefore, the reported rate of treatment-related deaths from the program-wide studies was approximately 0.8% (82/10,000). While a causal role of ipilimumab in these 82 deaths could not be ruled out, confounding factors could be identified in most of these cases.

5.1.11. *Clinical Efficacy:*

Ipilimumab prolonged survival in subjects with pre-treated and previously untreated advanced melanoma, based on results from 2 large, multinational, double-blind, Phase 3 studies (MDX010-20 and CA184024), supported by data from Phase 2 studies.

In prostate cancer, ipilimumab is being evaluated in Phase 1 and 2 studies, as well as in a randomized Phase 3 trial. Although sample sizes were small, response as measured by ≥50% decline in PSA have been reported. Responses were durable, ranging between 2 and 24 months.

*5.1.12. Association between safety (IRAEs) and efficacy (OS):*

Results from MDX010-20 suggested a tendency for improved OS in subjects with any IRAEs. In CA184024, analyses using the Cox proportional hazards model were conducted to assess the association of IRAEs and OS. Overall, the results showed a significant improvement in OS in subjects with Grade 3/4 IRAEs (any Grade 3/4 : HR 0.23 [95% CI: 0.10, 0.54]; liver Grade 3/4 : HR 0.25 [95% CI: 0.10, 0.65]). These results should be interpreted with caution, as the analysis was not adjusted for other prognostic factors.

Based on the current clinical experience with the use of corticosteroids for the management of treatment-emergent IRAEs, corticosteroids do not adversely affect the antitumor response in subjects with objective responses and concomitant serious IRAEs.

5.1.13. *Formulation*

Ipilimumab injection, 50mg/vial (5mg/mL) is formulated as a clear, colorless, sterile, non-pyrogenic, single-use, isotonic aqueous solution which may contain particles. It is supplied in 10-cc Type I flint glass vials stoppered with gray butyl stoppers and sealed with aluminum seals. The drug product is formulated at a concentration of 5mg/mL at a pH of about 7.

Each 50mg vial contains: 52.5mg Ipilimumab drug substance, 61.32mg sodium chloride USP, 33.10mg TRIS-hydrochloride, 0.4126mg diethylenetriamine pentacetic acid, 105mg mannitol USP, 1.05mg polysorbate 80 (plant-derived) 10.5mL water for injection USP qs to.

**Table 1: Ipilimumab Drug Information**

| Unit | Route | Appearance |
|---|---|---|
| Ipilimumab 5mg/ml, 10 mg or 40 mg vial | IV Infusion | Clear, colorless sterile solution in a single-use 10 ml or 40 mL vial |

*5.1.14. Packaging and Labeling*

Ipilimumab available at a concentration of 5 mg/mL in single use vials containing 10 ml (NDC 0003-2327-11) or 40 mL (NDC 0003-2328-22) solution.

*5.1.15. Storage, Handling and Dispensing of Ipilimumab*

1. *Storage*

   Ipilimumab should be stored in a secure area according to local regulations.

   The Investigator should ensure that the ipilimumab is stored in accordance with the environmental conditions (temperature, light and humidity) as determined by BMS and defined in the Investigator Brochure or SmPC/reference label.

   Ipilimumab must be stored at a temperature ≥ 2°C and ≤ 8°C. Do not freeze. Protect from light.

2. *Handling and Disposal*

   As with all, injectable drugs, care should be taken when handling and preparing ipilimumab. Whenever possible, ipilimumab should be prepared in a laminar flow hood or safety cabinet using standard precautions for the safe handling of intravenous agents applying aseptic technique.

   If ipilimumab concentrate or solution comes in contact with skin or mucosa, immediately and thoroughly wash with soap and water.

After final drug reconciliation, unused ipilimumab solution should be disposed at the site following procedures for the disposal of anticancer drugs.

3. *Dispensing*
   It is the responsibility of the Investigator to ensure that ipilimumab is only dispensed to study subjects. The ipilimumab must be dispensed only from official study sites by authorized personnel according to local regulations.

4. *Destruction of Ipilimumab:*
   If ipilimumab is to be destroyed on site, it is the Investigator's responsibility to ensure that arrangements have been made for disposal and that procedures for proper disposal have been established according to applicable regulations, guidelines, and institutional procedures. Appropriate records of the disposal must be maintained.

5. *Preparation of Ipilimumab*
   Ipilimumab injections will be prepared per the Package Insert.
   Do not draw into each vial more than once. Any partial vials should be safely discarded and should not be stored for reuse.

   Ipilimumab is administered as an IV infusion only.

6. *Administration*
   Ipilimumab will be administered as an IV infusion at doses of 1or 3 mg/kg dose over 90 (+/-10) minutes with a 10 cc normal saline flush at the end.

   ***<u>Dose Calculations</u>***

   • <u>Total dose</u> should be calculated as follows:
   Subject body weight in kg x 1-3 mg = total dose, mg

   • <u>Total infusion volume</u> should be calculated as follows:
   Total dose in mg ÷ 5 mg/mL = infusion volume, mL

   • <u>Rate of infusion</u> should be calculated as follows:
   Infusion volume in mL ÷ 90 minutes = rate of infusion, mL/min

   For example, a patient weighing 114 kg (250 lb) would be administered 1140 mg of ipilimumab (114 kg x 10 mg/kg = 1140 mg) with an infusion volume of 228 mL (1140 mg ÷ 5 mg/mL = 228 mL) at a rate of approximately 2.5 mL/min (228 mL ÷ 90 minutes) in 90 minutes.

## 5.2. Imatinib mesylate

5.2.1. *Physical/Chemical*
Imatinib mesylate is supplied commercially and additional information regarding the product may be obtained from the product's package insert.

- **Imatinib Mesylate:** (Gleevec, STI-571) manufactured by Novartis.
- **Chemical Name**: 4-[(4-Methyl-1-piperazinyl)methyl]-N-[4-methyl-3-[[4-(3-pyridinyl)-2-pyrimidinyl]amino]-phenyl]benzamide methanesulfonate.

- **Molecular Formula**: C30H35N7SO4 M.W.: 589.7CAS Registry Number: 220127-57-1
- **Approximate Solubility**: Imatinib mesylate is freely soluble in water and aqueous buffers < pH 5.0 bit is less soluble in neutral/alkaline aqueous buffers. In non-aqueous solvents, the drug substance is soluble in 1.2-propylene glycol, PEG 400, ethanol and methanol, but is poorly soluble in less polar solvents such as acetone and toluene.

- **Description**: Imatinib mesylate is a white to slightly yellowish crystalline powder.

5.2.2. *Mechanism of Action*:

In a pioneering work, Druker and co-workers demonstrated that imatinib mesylate suppressed proliferation of BCR-Abl-positive CML cells in vitro (12). Normal hematopoietic progenitors were largely unaffected (12). This compound was discovered to be an effective inhibitor of the PDGF receptor and KIT (CD 117, stem cell factor receptor) tyrosine kinases (9). Imatinib mesylate is specific with 50% inhibiting concentrations (IC50s) of 188nM for c-Abl, 413nM for KIT, 386nM for PDGFR-β. In contrast the IC50s of most of the other cellular tyrosine kinases (8, 10, 11) was found to be > 10 micromolar. These observations laid the groundwork for use of imatinib mesylate in the clinical setting, with potential for killing tumor cells harboring the target kinases without harm to normal host tissue. The antitumor effects of imatinib mesylate in GIST with activating kit mutations is remarkable (2, 3, 12-14).

Imatinib mesylate is a selective inhibitor of certain protein-tyrosine kinases. Imatinib mesylate inhibits the Abl tyrosine kinase as well as the KIT and PDGF-R RTKs. The compound specifically inhibits proliferation of v-Abl and BCR-Abl expressing cells, suggesting that it is not a general antimitotic agent. In colony formulation assays using ex vivo peripheral blood and bone marrow samples from patients with chronic myeloid leukemia (CML), imatinib mesylate shows selective inhibition of BCR-Abl positive colonies. In addition, imatinib mesylate is a potent inhibitor of receptors involved in both PDGF and SCF-mediated biochemical events (i.e., inhibiting both PDGF-R and KIT). In contrast, it does not affect signal transduction mediated by other stimuli including epidermal growth factor (EGF), insulin and phorbol esters. In vivo, the compound shows anti-tumor activity as a single agent in animal models as well tolerated doses.

5.2.3. *Pharmacology:*

Imatinib is prepared as a mesylate salt available in hard gelatin capsules that contain a common dry powder blend filled in capsule shells of size 100 mg 400 mg dosage strength tablets. It is the first of a class of drugs that act by specifically inhibiting receptor tyrosine kinases characteristic of a particular cancer cell, rather than non-specifically inhibiting and killing rapidly dividing cells.

Route of Administration: Oral. Imatinib mesylate should be taken with a meal to minimize GI irritation. Imatinib mesylate is a local irritant and must be taken in a sitting position with a large (250 ml; 8 oz) glass of water. (Direction of use on medication label: Take as directed with a large glass of water).

Supplier: Imatinib mesylate is commercially available and FDA approved for patients with advanced or metastatic GIST.

5.2.4. *Pre-clinical Toxicology:*

In in vitro human liver microsomes studies, imatinib mesylate appeared to be a competitive inhibitor of CYP2C9, CYP2D6, CYP3A4/5, suggesting that imatinib

mesylate could reduce the clearance of co-administered drugs whose metabolism is dependent on these P450 cytochrome isoenzymes.

Imatinib mesylate causes abortions and is potentially teratogenic at high doses in rabbits and rats. The compound is therefore not suitable for administration to pregnant women, and conception while on therapy should be avoided. In women of childbearing potential, contraception should continue for seven days after the last dose of imatinib mesylate to allow the complete clearance of drug and its principle metabolites from the body. Since interactions with the metabolism of oral contraceptives cannot be excluded at present, a barrier method of contraception must be used. Imatinib mesylate should not be administered to patients who are breastfeeding.

Imatinib mesylate was clastogenic according to the results of one of the genotoxicity tests performed. These effects were seen only at toxic concentrations and all other tests were negative; therefore, imatinib mesylate is not considered to present a genotoxic hazard.

Although there is no suggestion from available animal toxicology studies that imatinib mesylate enters the mammalian testis, this possibility cannot be excluded. Rats had reduced testis and epididymis weights and decreased sperm motility. Both male and female patients must continue to use effective methods of contraception for three months after receiving the last dose of study drug.

#### *5.2.5. Pharmacokinetics of Imatinib mesylate in Patients*

The pharmacokinetics of imatinib mesylate are similar in patients with CML and GIST (2). Imatinib mesylate has an oral bioavailability of > 97% in oral solution or capsule form (29). Once absorbed, it binds avidly to serum proteins and reaches peak concentrations in the serum 4 hours after administration (4-5 µg/mL for a 600-mg dose and 2-3 µg/mL for a 400-mg dose)(30). Imatinib mesylate crosses the blood-brain barrier and results in a 38 ng/mL concentration in the cerebral spinal fluid after a dose of 400-600 mg per day (30). Drug accumulation of 1.5-3 fold occurs after daily dosing, with a steady state reached within 1 week (31). Approximately 13% of the drug is excreted in the urine, while most is metabolized in the liver by the cytochrome P450 isoenzyme CYP3A4. Imatinib mesylate's major metabolite is N-desmethyl-imatinib (CGP74588), and its concentration is approximately 17% of imatinib mesylate's at steady-state conditions. This metabolite has been shown to have comparable activity to imatinib in vivo. The half-life of imatinib mesylate is approximately 25 hours, whereas that of its metabolite is 89 hours.

Because imatinib mesylate is hepatically metabolized by CYP3A4, drugs that are administered with it may undergo changes in their pharmacokinetics and vice versa. For example, ketoconazole, a broad-spectrum antifungal agent, was shown to increase patients' exposure to imatinib mesylate when co-administered (29). Additionally, rifampicin increased blood levels of imatinib mesylate. Conversely, imatinib mesylate increased the exposure of patients to simvastatin, a 3-hydroxy-3-methylglutaryl-coenzyme A (HMG-CoA) reductase inhibitor (32). Moreover, several other pharmaceuticals for cancer patients, such as alprazolam, caffeine, clindamycin, clonazepam, cortisol, ethinyl estradiol, and verapamil, may cause toxic effects when administered with imatinib mesylate (32). Frye et al recently published that the very popular over the counter product, St John's wort increased imatinib clearance by 43%

(33). Acetaminophen is also metabolized by CYP3A4 and patients should be advised to avoid daily use or excessive amounts of this agent. Recent studies have also shown in vitro synergism between imatinib mesylate, other tyrosine kinase inhibitors, and cytotoxic chemotherapeutics (34).

5.2.6. *Clinical Safety*

Treatment with imatinib mesylate was generally well-tolerated, although nearly every patient experienced at least some minor adverse events. The most frequently reported adverse events were edema, nausea, diarrhea, musculoskeletal pain, fatigue, rash, headache, and abdominal pain. Most events were of mild to moderate severity. Superficial edema, most frequently periorbital or lower limb edema, was managed with diuretics, other supportive measures, or by reducing the dose of imatinib mesylate. Severe (NCI CTCAE grade 3 or 4) superficial edema was observed in two patients, including facial edema in one patient. No major differences were seen in the severity of adverse events between the 400 mg or the 600 mg treatment groups, although overall incidence of adverse events was somewhat higher in the 600 mg treatment group. Adverse events with a suspected relationship to therapy occurring in >10% of patients in any group are presented in Table 2.

There was no hyperuricemia or evidence of tumor lysis syndrome, even in patients with very rapid decreases in tumor volume. The most medically significant adverse events were gastrointestinal or intra-abdominal hemorrhage in patients with large bulky tumors, which occurred in less than 5 % of patients. Studies in rats have shown that taking imatinib mesylate (usually at doses higher than that given to humans) may cause an increased risk of developing tumors, both benign and malignant. To date, there is no evidence that there is an increased risk in humans, but the possibility cannot be ruled out. Imatinib mesylate is indicated for the treatment of patients with KIT (CD117) positive unresectable and/or metastatic malignant (GIST).

The 2004 NCCN consensus guidelines for GIST and clinical experience states that patients who meet the criteria of disease progression may still be receiving benefit from imatinib therapy and should continue this agent. Discontinuation of imatinib may result in accelerated tumor growth (14).

**Table 2: Adverse Events Related to Imatinib Mesylate Therapy.**

| | All grades | | | Grade 3 / 4 | | |
|---|---|---|---|---|---|---|
| | 400 mg n = 73 | 600 mg n = 74 | All doses N = 147 | 400 mg n = 73 | 600 mg n = 74 | All doses N = 147 |
| Adverse Event | % | % | % | % | % | % |
| Any AE | 97 | 99 | 98 | 21 | 22 | 21 |
| Edema/fluid retention | 71 | 77 | 74 | 1 | 1 | 1 |
| Periorbital edema | 45 | 50 | 48 | 0 | 0 | 0 |
| Edema lower limb | 26 | 15 | 20 | 0 | 0 | 0 |
| Face edema | 8 | 12 | 10 | 1 | 0 | 1 |
| Edema | 7 | 14 | 10 | 0 | 0 | 0 |
| Eyelid edema | 7 | 8 | 8 | 0 | 0 | 0 |
| Nausea | 51 | 54 | 52 | 1 | 1 | 1 |
| Diarrhea | 40 | 50 | 45 | 1 | 3 | 2 |
| Myalgia / musculoskeletal pain | 37 | 42 | 40 | 0 | 0 | 0 |
| Fatigue | 30 | 39 | 35 | 0 | 0 | 0 |
| Dermatitis / rash | 25 | 37 | 31 | 3 | 3 | 3 |
| Headache | 19 | 32 | 26 | 0 | 0 | 0 |
| Abdominal pain | 26 | 26 | 26 | 1 | 0 | 1 |
| Flatulence | 19 | 24 | 22 | 0 | 0 | 0 |
| Vomiting | 14 | 12 | 13 | 0 | 1 | 1 |
| Any hemorrhage | 11 | 14 | 12 | 4 | 5 | 5 |
| Tumor hemorrhage | 1 | 4 | 3 | 1 | 4 | 3 |
| Upper GI bleed / perforation | 4 | 3 | 3 | 4 | 1 | 3 |
| Dyspepsia | 10 | 12 | 11 | 0 | 0 | 0 |
| Lacrimation increased | 7 | 12 | 10 | 0 | 0 | 0 |
| Anemia | 6 | 12 | 9 | 1 | 3 | 2 |
| Loose stools | 7 | 10 | 8 | 0 | 0 | 0 |
| Taste disturbance | 3 | 14 | 8 | 0 | 0 | 0 |

The US-Finland phase II trial demonstrated that imatinib mesylate was generally well tolerated. However, virtually every patient had at least some mild or moderate adverse events (grade 1 or 2) that were attributable to therapy (2). The most common adverse events were edema (which was most frequently periorbital), nausea, diarrhea, myalgia or musculoskeletal pain, fatigue, rash, headache, and abdominal pain (see Table 2). Although most of these adverse events were mild or moderate, 21% of patients had serious adverse events (grade 3 or 4). A few patients (5%) experienced intraabdominal hemorrhages (2), which were postulated to be associated with massive tumor necrosis induced by this active agent.

Early toxicity results of the large phase III trial conducted by the EORTC Soft Tissue and Bone Sarcoma Group, Italian Sarcoma Group, and Australasian Gastro-Intestinal

Trials Group were reported at the ASCO 2002 annual meeting. The most frequent side effects were anemia (88%), edema (67%), fatigue (60%), nausea (44%), neutropenia (32%), and skin rash (24%). Most side effects were mild to moderate; however, one patient died of drug-related neutropenic sepsis (35). In summary, imatinib mesylate is safe and generally well tolerated at doses up to 800 mg daily.

5.2.7. *Clinical Efficacy:*

The first patient with metastatic GIST was treated at the University Hospital of Helsinki, Finland with imatinib mesylate at 400 mg/day. The first dose was administered on 07-Mar-00. After four weeks, a first response to treatment was reported in the form of an approximately 40-50% decrease in the size of the largest liver lesion. After eight weeks of treatment, MRI confirmed a partial response. In addition, histological and immunohistochemial findings were consistent with a marked reduction if KIT positive cells, showed the replacement of tumor tissue by necrotic and fibrotic tissue, and marked reduction in fluorodeoxyglucose uptake of all metastases was seen in serial PET scans (36).

An open-label, multinational study was conducted in patients with unresectable or metastatic malignant GIST (2). In this study, 147 patients were enrolled and randomized to receive either 400 mg or 600 mg orally daily for up to 24 months. Patients ranged in age from 18-83 years old and had a pathologic diagnosis of Kit-positive unresectable and/or metastatic malignant GIST. The primary outcome of the study was objective response rate. Tumors were required to be measurable at entry in at least one site of disease, and response characterization was based on Southwestern Oncology Group (SWOG) criteria. All patients have had greater than nine months of follow-up. The median time to objective response was 13 weeks. Objective response rate was 54% with no complete responses. Twenty-eight percent of patients had stable disease; 14% of patients progressed, and 6% of patients were not evaluable. Reduction in tumor bulk for patients achieving a PR ranged from 50-96%. Responses have been durable for more than 46 weeks and median duration of response has not yet been reached (median follow-up: 24 weeks following onset of response). ECOG performance scores (PS) improved with imatinib mesylate therapy consistent with objective antitumor activity. By month 4 of the study, the number of patients with normal functional status (PS=0) increased to 64% from 42% at study entry. This study was updated at ASCO 2002 and confirmed PR response rates were 63%; 19% of patients had stable disease.

1. Phase I studies of Imatinib Mesylate in Gastrointestinal Stromal Tumor:

A single-patient pilot study confirmed the efficacy of imatinib mesylate in GIST. This first patient to be treated with imatinib mesylate was a 50-year-old woman with chemotherapy-resistant, metastatic GIST who received once-daily doses of 400 mg of imatinib mesylate starting in March 2000. Response was evaluated objectively, using 18-fluorodeoxyglucose-positron emission tomography (PET) and computed tomographic radiography. The patient's tumor remained stable after a year of therapy, and she had only mild GI side effects. Serial tumor biopsies revealed myxoid degeneration after only 4 weeks of treatment (36).

A phase I study of imatinib mesylate in GIST was done in three centers of the European Organization for Research and Treatment of Cancer (EORTC) Soft Tissue and Bone Sarcoma Group (14). Between August 3, 2000, and December 21, 2000, 40 patients (36 patients with advanced GIST) received imatinib mesylate at doses of 400

mg once daily, 300 mg twice daily, 400 mg twice daily, or 500 mg twice daily. The maximum tolerated dose of imatinib mesylate was judged to be 400 mg twice daily, owing to unacceptable toxicity at the 500-mg, twice daily dose, which included grade 3 nausea/vomiting, edema, and dyspnea. Myelosuppression was an infrequent side effect and did not seem to be dose-dependent. However, mild anemia and neutropenia grade 2 or 3 were reported. Although not the primary endpoint, a partial response rate of 53% was reported (14).

2. Phase II studies of imatinib mesylate in Gastrointestinal Stromal Tumor:

   These encouraging results, as well as the experience of using imatinib mesylate in patients with CML, led to the rapid deployment of several phase II and phase III studies of imatinib mesylate in GIST. The initial trial, designated as the US-Finland trial (2), was a multicenter, open-label, randomized phase II clinical trial of imatinib mesylate in patients with unresectable or metastatic, KIT-expressing GIST. Between July 2000 and April 2001, 147 patients were randomly assigned to receive 400 or 600 mg of imatinib mesylate orally daily. At a median follow-up of 24 months, 63% of patients in the US-Finland trial had a partial response, 19% of patients had stable disease, and 12% had confirmed tumor progression. The median time to progression was 72 weeks, and the median survival had yet to be reached. The response rates did not differ significantly between the two doses (2).

   The above results were confirmed with another phase II trial performed by the EORTC Soft Tissue and Bone Sarcoma Group. A total of 27 patients with advanced and/or metastatic GIST received imatinib at the highest feasible dose of 400 mg twice daily. Side effects were mild to moderate, and the most common included anemia, periorbital edema, skin rash, fatigue, nausea, granulocytopenia, and diarrhea. Response rates were similar to those in the US-Finland phase II trial: 4% complete response, 67% partial response, 18% stable disease, and 11% disease progression. At 1 year, 73% of patients were free from disease progression (37)).

3. Phase III studies of imatinib mesylate in Gastrointestinal Stromal Tumor:

   Two large consortia conducted two phase III studies nearly simultaneously. One was the North American Sarcoma Intergroup study S0033, consisting of the US cooperative oncology groups (Southwest Oncology Group, Cancer and Leukemia Group B, and the Eastern Cooperative Group) as well as the National Cancer Institute of Canada Sarcoma Group. The primary aim of this study was to assess the impact of imatinib mesylate dose (400 mg vs. 800 mg daily) on survival; secondary aims were to evaluate response rates and confirm the tolerability of imatinib mesylate therapy in patients with GIST. Between December 15, 2000, and September 1, 2001, 746 patients from 57 institutions were enrolled. Patients randomized to receive the 400-mg daily dose were allowed to cross over to the 800-mg daily dose if they had progressive disease. At a median follow-up of 14 months, overall response rates were similar in both arms: 43% at the 400-mg dose and 41% at the 800-mg dose (3). There was no difference in progression-free or overall survival between dose levels. Median overall survival had not been reached in either arm after a median follow-up of 25.6 months, and there continued to be no significant differences between the two arms in regards to progression-free and overall survival. Progression-free survival rate estimates at 2 years are 50% vs. 53% for the 400-mg vs. 800-mg arms, respectively. Survival estimates at 2 years are 78% vs. 73% for the 400-mg vs. 800-mg arms, respectively. However, of the 106 patients who crossed over to the higher dose after having

progressive disease on the 400-mg daily dose, 7% had a partial response and 32% had stable disease, indicating that patients can benefit from a higher dose after their disease progresses on 400 mg daily.

The EORTC Soft Tissue and Bone Sarcoma Group, Italian Sarcoma Group, and Australasian Gastro-Intestinal Trials Group conducted the second phase III trial of imatinib mesylate. Between February 2001 and February 2002, 946 patients with GIST were randomized to receive imatinib mesylate at a dose of either 400 mg daily or 400 mg twice daily. This trial was powered to detect a 10% difference in progression-free survival rates, with objective response to treatment as a secondary endpoint. The objective response rates were 50% vs. 54% for the 400-mg vs. 800 mg arms, respectively. The two-year overall survival estimate was 69% for patients treated at an initial daily dose of 400-mg and 74% for those patients started at 400-mg twice daily (p not significant). Progression-free survival was 44% vs. 52% (p=0.026) for patients allocated to imatinib once a day compared to twice a day, respectively (37). As reported earlier, the North American trial did not show a difference in survival or progression-free survival, and the reason for this discrepancy is unknown. It is possible that different results in the two studies is due to the greater number of patients enrolled in the EORTC study allowing more power to detect statistical differences. Another possible explanation would be different genetic composition of patients enrolled in the two trials. Moreover, the patients enrolled in these two trials have not been analyzed by location of kit mutation. It is possible that exon 11 mutation was more common in tumors from patients enrolled in the EORTC study as compared to the U. S. Intergroup trial.

5.2.8. *Formulation*

Gleevec film-coated tablets contain Imatinib mesylate, which is a white to off-white to brownish or yellow tinged crystalline powder, equivalent to 100 mg or 400 mg imatinib free base. The film-coated tablets are ovaloid, biconvex with beveled edges, debossed with “NVR” on one side with a “SA” on the other side (for 100 mg tablets) or “400” on one side and score on the other side with “SL” on each side of the score.

Inactive Ingredients include: colloidal silicon dioxide (NF); crospovidone (NF); hydroxypropyl methylcellulose (USP); magnesium stearate (NF); and microcrystalline cellulose (NF).

Tablet Coating: ferric oxide, red (NF); ferric oxide, yellow (NF); hydroxypropyl methylcellulose (USP); polyethylene glycol (NF) and talc (USP).

5.2.9. *Packaging and Labeling*

Imatinib mesylate is available in 90 tablets (NDC 0078-0401-34) or 30 tablets per bottle (NDC 0078-0438-15).

5.2.10. *Storage, Handeling and Despensing of Imatinib mesylate*

- Store at 25°C (77°F); excursions permitted to 15-30°C (59-86°F) [see USP Controlled Room Temperature].
- Protect from moisture.
- Dispense in a tight containers, USP.
- Procedures for proper handling and disposal of anticancer drugs should be considered. Several guidelines on this subject have been published (38-41).

5.2.11. *Preparation of Imatinib mesylate*

Imatinib mesylate will be manufactured, prepared, and dispensed by Novartis.

5.2.12. *Administration:*

Imatinib mesylate will be administered orally as 400 mg tablets either once or twice per day, depending on dosage scheme described below. Imatinib mesylate should be taken with a meal to minimize GI irritation. Imatinib mesylate is a local irritant and must be taken in a sitting position with a large (250 ml; 8 oz) glass of water. (Direction of use on medication label: Take as directed with a large glass of water).

# 6. Treatment Plan

Notice: Treatment schedules shall have a standing window of allowance of +/- 2 days. Any treatment day that falls on a weekend or holiday will be scheduled on the next business day. Patients will undergo a therapy wash out period of 5 drug half-lives or 4-weeks and radiation therapy wash out of 2-weeks prior to study enrollment.

For treatment or dose modification questions, please contact David Hong, MD, by phone (713-563-5844) or e-mail (dshong@mdanderson.org). No investigational or commercial agents or therapies other than those described below may be administered with the intent to treat the patient's malignancy.

**6.1. Agent Administration and Dose Escalation**

The dose escalation portion of this study will consist of an initial daily oral administration of imatinib mesylate alone for 14 days (14-day run in; before Cycle 1 only). A single Ipilimumab treatment given on day 15 (42) will be added to daily imatinib mesylate therapy. This study has an expansion cohort using the MTD determined by the dose escalation study to treat patients with *KIT* confirmed GIST, melanoma, and uncategorized solid tumors. Both studies will consist of a screening visit and continuous 21-day treatment cycles. Cycles will be repeated every 21 days for 4 cycles until disease progression or development of intolerable toxicities, and a post-treatment visit (42). Prior to start each treatment cycle, patients will arrive at the Clinical Center for Targeted Therapy and undergo safety assessments. Patients will return to the study center for a post-treatment visit within 21 days (± 2) from the date of last dose ipilimumab. In both arms, safety will be assessed by physical examination, observing and questioning patients regarding adverse experiences, and monitoring clinical chemistry and hematology. Disease progression will be based upon the irRC Criteria. To be considered evaluable, patients must meet eligibility criteria and complete at least 1 treatment cycle. Patients who withdraw from the imatinib alone due to toxicity or progressive disease prior to completing the first cycle disease assessment will not be evaluable. Patients who are not considered evaluable will be replaced.

6.1.1. *3+3 Dose Escalation of Ipilimumab and Imatinib mesylate*

This study will be broken into 4 cohorts of 3 patients each cohort. The first cohort of patients will receive the lowest dosing regimen (1) as outlined below (Table 3), with subsequent cohorts receiving increasing doses of ipilimumab and/or imatinib mesylate until DLT are

seen and MTD determined.

Patients will receive a 14-day run in therapy of daily imatinib mesylate dose before cycle 1 only (see dose escalation scheme and Figure 1). If patient has ≥ Grade 3 toxicities, they will be dropped and replaced for this study. If patient experiences ≤ Grade 3 toxicity, they will proceed into cycle 1 of ipilimumab/imatinib combination therapy. Each patient will receive 2 cycles of ipilimumab and imatinib mesylation combination therapy and assessed for DLT at day 35 (± 2) and will be restaged at the end of Cycle 2. If patients show no DLTs, stable disease, partially responsive disease, or non-progressive disease, they will continue for upto 4 doses ipilimumab and daily imatinib mesylated therapy. After cycle 4 of this study, patients will be again assessed for AEs and restaged. If patients still show, stable disease, partially responsive disease, or non-progressive disease, they will continue with daily imatinib mesylate therapy. Patients will be assessed for response criteria every 2 cycles.

**Table 3: Dose Escalation**

| Dose Level | Imatinib Mesylate | Ipilimumab |
|---|---|---|
| -1 | 400 mg once per day for 35 days | 0.5 mg/kg on day 15 |
| 1 | 400 mg once per day for 35 days | 1 mg/kg on day 15 |
| 2 | 400 mg twice per day for 35 days | 1 mg/kg on day 15 |
| 3 | 400 mg once per day for 35 days | 3mg/kg on day 15 |
| 4 | 400 mg twice per day for 35 days | 3mg/kg on day 15 |

**Table 4: Regimen Description**

| Agent | Premedications; Precautions | Dose | Route | Schedule | Cycle Length |
|---|---|---|---|---|---|
| Imatinib mesylate (Gleevec) | None | Number of 400 mg tablets depending on dose level | Oral | Daily alone: Days 1-14 Combination: Days 15-35 | 21 days (3 weeks; ± 2 days) |
| Ipilimumab (Yervoy) | None | Mix in 100 mL of 0.9% Normal Saline | IV 90 minute infusion, reducing as possible | Day 15 (± 2) | |

**1. Imatinib mesylate (Gleevec)**

Imatinib mesylate 400 mg (number of tablets determined by dosing regimen) tablets will be taken orally for 14 days (± 2 days) prior to first ipilimumab administration (14-day run in before Cycle 1 at dose escalation only) and 21 days (± 2 days) as combination therapy with ipilimumab. If patient experience a Grade ≥ 3 toxicity related to imatinib mesylate during the two week run in before ipilimumab treatment, the patient will be dropped and replaced for

that cohort. All study participants will record each dose in a diary.

Imatinib mesylate should be taken with a meal to minimize GI irritation. Imatinib mesylate can be a local irritant and must be taken in an upright position with at least 8 ounces of water. Grapefruit or grapefruit juice should not be ingested while on treatment with imatinib mesylate. Patients should avoid drinking beverages with caffeine within one hour of taking imatinib mesylate.

Patients taking acetaminophen (Tylenol) while on imatinib mesylate may be at a greater risk for liver or kidney damage. It is recommended that no more than 500 mg of acetaminophen be taken every 6 hours. All over the counter and prescription drugs should be carefully reviewed as these drugs could possibly contain acetaminophen.

**2. Imipimumab (Yervoy)**
Ipilimumab will be administered as a single 90 minute (+/- 15 minutes) intravenous infusion on day 15 (± 2). The calculated dose will be diluted in 100 mL of 0.9% Normal Saline.

Ipilimumab and imatinib mesylate combination therapy will be completed for 4 x 21 day (± 2) cycles.

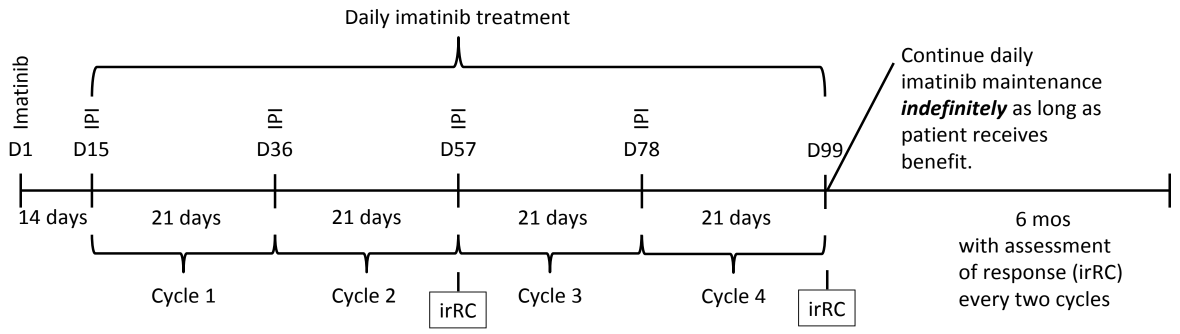


**Figure 1: Diagram of Treatment Schedule for Imatinib Mesylate and Ipilimumab Combination Dose Escalation Study.**

6.1.2. *Expansion Cohort: Combination Therapy in Patients with GIST and KIT Confirmed Advanced Malignancies with Ipilimumab and Imatinib mesylate combined MTD*

After the MTD has been determined, this dosage scheme will be used to treat patients with *KIT* confirmed GIST, melanoma, and other uncategorized solid tumors (Table 5). *KIT* positive tumors will be confirmed in previously biopsied tumors and biopsied tumors acquired during this study. This analysis will be done by CLIA approved mutational analysis which will likely include isolation of genomic DNA from tumor, polymerase chain reaction (PCR) amplification of exons 8, 9, 11, 13, and 17 of c-*KIT* gene (regions previously been reported to carry gain-of-function *KIT* mutations (16, 18, 43, 44), bidirectional sequencing of PCR amplicons, and computational analysis of sequences to determine mutation presence or absence. Any tumors harboring mutations or amplifications found within exons 8, 9, 11, 13, and/or 17 that result in any change in amino acid sequence, which include the most commonly found *KIT* mutations within the juxtamembrane domain of c-*KIT (16, 18, 43, 44)*, will be considered positive *KIT* tumors. Tumor samples that show c-KIT expression in by immunohistochemistry will also be accepted. These patients will be treated using the treatment regimen outlined in Table 5 for 4 x 21 day cycles (Figure 2).

**Table 5: Effectiveness of ipilimumab and Imatinib mesylate Combination Therapy**

| Patient Selection | Dose: Ipilimumab/Imatinib Combination Therapy |
|---|---|
| Patients with *KIT* GIST Tumors | MTD |
| Patients with *KIT* confirmed melanoma | MTD |
| Patients with other *KIT* confirmed solid tumors | MTD |

**Table 6: Regimen Description: Expansion Cohort**

| **Agent** | **Premedications; Precautions** | **Dose** | **Route** | **Schedule** | **Cycle Length** |
|---|---|---|---|---|---|
| Imatinib mesylate (Gleevec) | None | ** MTD in Number of 400 mg tablets | Oral | Daily: Days 1-21 | 21 days (3 weeks) |
| Ipilimumab (Yervoy) | None | ** MTD mixed in 100 mL of 0.9% Normal Saline | IV 90 minute infusion | Day 1 (+ 2) | |

** Doses as appropriate for assigned MTD dose level.

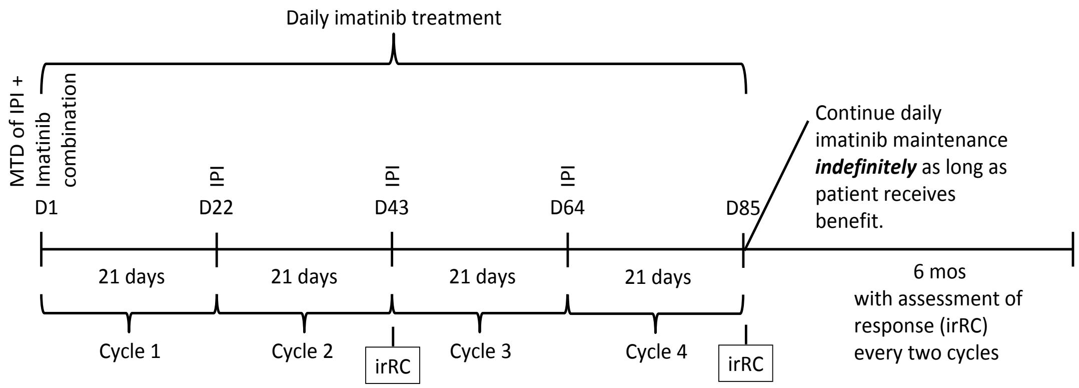


**Figure 2: Diagram of Treatment Schedule for Expansion Cohort using MTD for Imatinib Mesylate and Ipilimumab Combination Therapy in *KIT* Confirmed GIST, Melanoma, and Uncategorized Solid Tumors.**

### 6.2. Definition of Dose-Limiting Toxicity and Maximum Tolerated Dose:

6.2.1. *Dose Limiting Toxicity (DLT)*:

**DLT is defined as any:**

- Any adverse event (AE) of severity grade 3 or 4 (including serious or life-threatening) considered possibly, probably or definitely related to ipilimumab and/or imatinib (CTCAE v4.0: Excluding those events that occur and are completely resolved within 4-6 hours of the first dose of imatinib or ipilimumab (infusion-related reactions),
- Any clinically grade 3 or 4 non-hematologic toxicity as defined in the NCI CTC v4.0, expected and believed to be related to the study medications (except nausea and vomiting, diarrhea and electrolyte imbalances responsive to appropriate regimens, alopecia or fatigue lasting less than 7 days)
- Any grade 4 neutropenia (with or without fever and/or sepsis) or thrombocytopenia (with or without bleeding) lasting at least 1 week or longer (as defined by the NCI-CTC v4.0)
- Any of the Grade 4 hematologic adverse events for >5 days
- Any grade 3 or 4 nausea or vomiting lasting more than 5 days despite anti-emetics regimens or grade 3 or 4 diarrhea refractory to anti-diarrhea medications
- Grade 3 fatigue lasting more than 7 days
- Any other grade 3 non-hematologic toxicity including symptoms/signs of vascular leak or cytokine release syndrome; or any severe or life-threatening complication or abnormality not defined in the NCI-CTCAE v4.0 that is attributable to the therapy

**The Maximum Tolerated Dose (MTD) is defined:**

- The highest dose level with less than 2 patients with DLT out of at least six patients in the cohort. Management and dose modifications associated with adverse events are outlined in below table.

| **Number of Patients with DLT* at a Given Dose Level** | **Escalation Decision Rule** |
|---|---|
| 0 out of 3 | Enter 3 patients at the next dose level. |
| ≥2 | Dose escalation will be stopped. This dose level will be declared the maximally administered dose (highest dose administered). Three (3) additional patients will be entered at the next lowest dose level if only 3 patients were treated previously at that dose. |

| | |
|---|---|
| 1 out of 3 | Enter at least 3 more patients at this dose level.<br><br>If 0 of these 3 patients experience DLT, proceed to the next dose level.<br><br>If 1 or more of this group suffer DLT, then dose escalation is stopped, and this dose is declared the maximally administered dose. Three (3) additional patients will be entered at the next lowest dose level if only 3 patients were treated previously at that dose. |
| < =1 out of 6 at highest dose level below the maximally administered dose | This is generally the MTD. At least 6 patients must be entered at the MTD. |

***The time window for DLT evaluation in the dose escalation phase is 35 days (14 days of daily imatinib mesylate alone and 21 days of ipilimumab/imatinib combination therapy*)

If a response has been observed in a particular tumor type with the study drug or drug combination, then the study may be expanded to include up to 3 additional participants with that tumor type. All patients will be treated at the highest current dose level. All enrolled participants will be considered in the DLT analysis. ***If at any time more than or equal to one third of the participants at a dose level experience DLT, the MTD will be reassessed and the next lowest dose level for the combination therapy will be considered the MTD.*** Patients experiencing DLTs at a specific dose will be allowed to continue combination therapy at the next lowest dose. If patients cannot tolerate either of the drugs, they will be allowed to continue treatment of single drugs with the discretion of the physician. For the purpose of adding up to 3 additional participants, a tumor response is defined as one or more of the following: (1) stable disease for more than or equal to 4 months, (2) decrease in measurable tumor (sentinel lesions) by more than or equal to 20% by irRC criteria.

**6.3. Duration of Therapy and Criteria for Treatment Delay**

6.3.1. *Duration of Therapy:*

In the absence of treatment delays due to adverse events, study treatment will continue until day 35 (14 days of daily imatinib mesylate and 21 days of ipilimumab/imatinib combination therapy) as described in 6.1.1. If no DLTs are experienced and disease has not progressed, combination therapy will continue for a totally of 4 cycles followed by a daily maintenance dose of imatinib mesylate.

6.3.2. *Criteria for Treatment Delay:*

Patients will delay treatment with ipilimumab or combination therapy if they experience at least one of the following adverse events considered by the Investigator to be “possibly”, probably” or “certainly” related to ipilimumab treatment:

- Any Grade 3 non-skin related adverse event (excluding alopecia, and Grade 3 nausea, vomiting, and diarrhea for which adequate supportive therapy has been instituted)
- Any Grade 3 skin-related adverse event (including irAEs)
- Any of the Grade 4 hematologic adverse events for >5 days
- Grade 3 fatigue only if ≥ 7 days

6.3.3. *Criteria for Restart of Treatment:*

Patients experiencing DLTs at a specific dose will be allowed to continue combination therapy at the next lowest dose. Treatment with ipilimumab combination therapy can be restarted as long as:

- The adverse event is not listed in 6.2.1, and
- If the adverse event has resolved to ≤ Grade 1 severity or returns to baseline within 4 weeks (28 days) of dose administration, ipilimumab and Imatinib mesylate will be restarted ≥4 weeks from the last dose administration, to complete dosing regimen outlined above.
- If the adverse event has not resolved to ≤ Grade 1 severity or returned to baseline in the protocol-specified dosing window (3 weeks), the next scheduled dose will be omitted and remaining doses of ipilimumab administered if approved by the Principal Investigator.
- Treatment delays for reasons other than adverse events (e.g. for scheduling conflicts) are allowed as long as all four ipilimumab doses are given within 8 months. In this scenario, all 4 doses should be administered separated by a minimum interval of 3 weeks.

6.3.4. *Criteria for Permanent Discontinuation of Ipilimumab*

- Patients will remain on study (and thus, continue imatinib mesylate treatment as described in 5.1) but will receive no further treatment with ipilimumab if:
- They suffer any of the following adverse events with at least a possible, probable or definite attribution to Ipilimumab:
    - Any ≥ Grade 3 eye pain or reduction of visual acuity which:
        - o does not respond to topical therapy and improves to ≤ Grade 1 severity within 2 weeks of starting therapy, OR,
        - o requires systemic treatment;
    - Any ≥ Grade 3 bronchospasm or other hypersensitivity reaction;
    - Any ≥ Grade 3 immune-related AE with the exception of those listed under section 6.3.2.
    - Any other ≥ Grade 4 non-skin related adverse event.
    - Any clinical adverse event, laboratory abnormality or intercurrent illness which requires ongoing treatment with systemic corticosteroids and/or other systemic immunosuppressants (except if the patient is receiving stable doses of imatinib mesylate).
    - Any clinical adverse event, laboratory abnormality or intercurrent illness which, in the opinion of the Investigator, indicates that continued treatment with ipilimumab is not in the best interest of the subject.
    - Hospitalization for a serious adverse event related to study drug.

1. *Exceptions to Permanent Discontinuation of ipilimumab dosing:*
- Hospitalization for ≤ Grade 2 adverse events where the primary reason for hospitalization is to expedite the clinical work-up;

6.3.5 *Criteria for Removal from Study*

Subjects MUST be discontinued from study therapy AND withdrawn from the study for the following reasons:

- Progression of disease per imaging criteria as described previously (Exception: If the patient is deriving clinical benefit from the treatment, then the patient may continue on study at the discretion of the PI). (see section 7.5.1 for definition of disease progression according to irRC).
- The development of unacceptable toxicity
- Pregnancy
- Any other situation where, in the opinion of the treating physician, continued treatment per protocol, would not be in the best interest of the patient.
- The patient withdraws consent (subject's decision to withdraw for any reason).
- Imprisonment or the compulsory detention for treatment of either a psychiatric or physical (e.g., infectious disease) illness.
- Study completion

NOTE: If patients cannot tolerate either of they drugs, they will be allowed to continue treatment of single drugs with the discretion of the physician. They will still be considered evaluable if they complete at least one cycle of combination therapy.

**6.4. Immune Related Adverse Events (IRAEs): Definition, Monitoring and Treatment**

Blocking CTLA-4 function may permit the emergence of auto-reactive T cells and resultant clinical autoimmunity. Rash/vitiligo, diarrhea/colitis, uveitis/episcleritis, hepatitis and hypopituitarism were drug-related, presumptive autoimmune events, now termed IRAEs, noted in previous ipilimumab studies.

For the purposes of this study, an IRAE is defined as an AE of unknown etiology, associated with drug exposure and is consistent with an immune phenomenon. Efforts should be made to rule out neoplastic, infectious, metabolic, toxin or other etiologic causes prior to labeling an AE an IRAE. Serological, immunological, and histological (biopsy) data should be used to support the diagnosis of an immune-mediated toxicity. Suspected IRAEs must be documented on an AE or SAE form.

Patients should be informed of and carefully monitored for evidence of clinically significant systemic IRAE (e.g., systemic lupus erythematosus-like diseases) or organ specific IRAE (e.g., rash, colitis, uveitis, hepatitis or thyroid disease). If an IRAE is noted, appropriate work-up (including biopsy if possible) should be performed, and steroid therapy may be considered if clinically necessary.

It has been reported that systemic corticosteroid therapy does not seem to have an attenuating effect on ipilimumab activity (Chen B; 98$^{th}$ AACR April 2007, abst 2202; Weber JS Melanoma Res 2006). However, administration of a prophylactic corticosteroid, budesonide, did not impart any clinical benefit in patients treated with ipilimumab (Weber J,

J Clin Oncol 2008). If utilized, corticosteroid therapy should be individualized for each patient. Prior experience suggests that colitis manifested as ≥ Grade 3 diarrhea requires corticosteroid treatment.

## 6.5. Infusion Reactions and Fever Associated with Ipilimumab

6.5.1. Infusion Reactions
Since ipilimumab contains only human protein sequences, it is less likely that any allergic reaction will be seen in patients. However, it is possible that infusion of ipilimumab will induce a cytokine release syndrome that could be evidenced by fever, chills, rigors, rash, pruritus, hypo- or hypertension, bronchospasm or other symptoms. No prophylactic pre medication will be given unless indicated by previous experience in an individual patient.

The following treatment guidelines are suggested:

1. **For MILD SYMPTOMS** (e.g., localized cutaneous reactions such as mild pruritus, flushing, rash):

- Decrease the rate of infusion until recovery from symptoms, remain at bedside and monitor patient;
- Complete the ipilimumab infusion at the initial planned rate ;
- Diphenhydramine 50 mg IV may be administered at the discretion of the treating physician and patients may receive additional doses with close monitoring;
- Premedication with diphenhydramine may be given at the discretion of the Investigator for subsequent doses of ipilimumab.

2. **For MODERATE SYMPTOMS** (any symptom not listed above [mild symptoms] or below [severe symptoms] such as generalized pruritus, flushing, rash, dyspnea, hypotension with systolic BP >80 mmHg):

- Interrupt ipilimumab;
- Administer diphenhydramine 50 mg IV;
- Monitor patient closely until resolution of symptoms;
- Corticosteroids may be administered at the discretion of the treating physician;
- Resume ipilimumab infusion after recovery of symptoms;
- At the discretion of the treating physician, ipilimumab infusion may be resumed at one half the initial infusion rate, then increased incrementally to the initial infusion rate.
- If symptoms develop after resumption of the infusion, the infusion should be discontinued and no additional ipilimumab should be administered that day;
- The next dose of ipilimumab will be administered at its next scheduled time and may be given with pre-medication (diphenhydramine and acetaminophen) and careful monitoring, following the same treatment guidelines outlined above;
- At the discretion of the treating physician additional oral or IV antihistamine may be administered prior to dosing with ipilimumab.

3. **<u>For SEVERE SYMPTOMS</u>** (e.g., any reaction such as bronchospasm, generalized urticaria, systolic blood pressure <80 mm Hg, or angioedema):

- Immediately discontinue infusion of ipilimumab, and disconnect infusion tubing from the subject;
- Consider bronchodilators, epinephrine 1 mg IV or subcutaneously, and/or diphenhydramine 50 mg IV, with solumedrol 100 mg IV, as needed.
- Patients should be monitored until the Investigator is comfortable that the symptoms will not recur;
- No further ipilimumab will be administered;

Note: In case of late-occurring hypersensitivity symptoms (e.g., appearance within one week after treatment of a localized or generalized pruritus), symptomatic treatment may be given (e.g., oral antihistamine, or corticosteroids).

### 6.6. Treatment of Ipilimumab Related Isolated Drug Fever

In the event of isolated drug fever, the Investigator must use clinical judgment to determine if the fever is related to the ipilimumab or to an infectious etiology.

If a patient experiences isolated drug fever, for the next dose, pre-treatment with acetaminophen or non-steroidal anti-inflammatory agent (Investigator discretion) should be instituted and a repeated antipyretic dose at 6 and 12 hours after ipilimumab infusion should be administered. The infusion rate will remain unchanged for future doses.

If a patient experiences recurrent isolated drug fever following pre-medication and post dosing with an appropriate antipyretic, the infusion rate for subsequent dosing should be decreased to 50% of the previous rate. If fever recurs following infusion rate change, the Investigator should assess the patient's level of discomfort with the event and use clinical judgment to determine if the patient should receive further ipilimumab.

### 6.7. Concomitant, Prohibited and Restricted Therapies During the Study

6.7.1. *Concomitant Therapies*

- Antiemetic Prophylaxis will be used to manage imatinib mesylate induced vomiting and nausea.
    - All patients should have antiemetic medications available once discharged from the clinic. Oral antiemetic medications should be prescribed and administered as needed, and adjusted during the cycle at the discretion of the treating investigator.
    - If patients experience nausea and vomiting despite the premedication, the patient may take PRN antiemetics per treating physician's discretion.
- Palliative radiation will also be allowed to treat any pain or discomfort resulting form tumor.

6.7.2. *Diarrhea Management for imatinib mesylate*

- All patients should be instructed to take loperamide at the earliest sign of diarrhea and/or abdominal cramping. These signs can include: a) loose stool, b) the occurrence of 1 to 2 more bowel movements than usual in 1 day, or c) an unusually high volume of stool.
- Loperamide should be dosed in the following manner: 4 mg at the first onset of

diarrhea, then 2 mg every 2 hours around the clock until diarrhea free for at least 12 hours. Patients may take Loperamide 4 mg every 4 hours during the night.

Note: Because there is a potential for interaction of imatinib mesylate with other concomitantly administered drugs through the cytochrome P450 system, the concurrent use of all other drugs, over-the-counter medications, or alternative therapies will be documented.

6.7.3. *Prohibited Therapies*
Patients in this study may not use vaccines for the treatment of cancer or prevention of disease for up to one month pre and post dosing with ipilimumab.

Concomitant systemic or local anti-cancer medications or treatments are prohibited in this study while receiving ipilimumab treatments, other than palliative (pain controlling) radiation therapy (RT) in situations that are not clearly indicative for PD.

Patients may not use any of the following therapies during the study:

- Any non-study anti-cancer agent (investigational or non-investigational);
- Any other investigational agents;
- Any other (non-CA184024 related) CTLA-4 inhibitors or agonists;
- CD137 agonists;
- Immunosuppressive agents;
- Chronic systemic corticosteroids;
- Any non-oncology vaccine therapies used for the prevention of infectious diseases (for up to 30 days prior to or after any dose of study drug).

# 7. Criteria for Evaluation

## 7.1. Evaluations at Baseline

Notice: On-study tests/visits that must occur within a defined time frame shall have a standing window of allowance that is equal to +/- 2 days for any laboratory testing.

7.1.1. *Four weeks prior to study initiation*

The following appropriate imaging studies for tumor assessment should be obtained within 4-weeks prior to study initiation.

- Determination and measurements of target lesions
- Contrast CT scans of the abdomen and pelvis (preferred) or MRI of abdomen and pelvis
- Chest X-ray (if lung metastases are evident on chest x-ray, CT of the chest should be obtained as well)
- Bone scan

7.1.2. *Two weeks prior to study initiation*

The following studies should be obtained within 2-weeks prior to study initiation.

- Electrocardiogram (ECG)

- Optional biopsies for expansion cohort initiation (financial support for biopsy cost to be determined).

7.1.3. *7-days prior to study initiation*.

The following studies should be obtained within 7 days prior to study initiation. All abnormal and normal results must be noted in the case report forms (CRF).

- Medical history to include determination of tumor-related symptoms.
- Physical examination to include height, weight and vital signs
- CBC with differential and platelet count.
- Serum chemistry studies to include sodium, potassium, chloride, carbon dioxide, BUN, creatinine, glucose, magnesium, calcium, phosphorus, albumin, total protein, SGOT or SGPT, total bilirubin, alkaline phosphatase, cholesterol, triglycerides and uric acid.
- Routine urinalysis.
- Serum pregnancy test for females of childbearing potential within 7 days of registration.
- Initial optional serum biomarkers will be obtained:
    - within 7-days prior to study initiation and (dose escalation and expansion cohorts)
    - right before first ipilimumab administration (dose escalation only)

### 7.2. Evaluations During Study

7.2.1. *Before each Ipilimumab administration*
Every patient needs to be evaluated by the physician before each ipilimumab administration

7.2.2 *Two-three weeks after first dose of ipilimumab is administered*

- Physical examination to include height, weight and vital signs.
- Physical examination, interim history pertaining to any change from baseline, current medications and treatment-related toxicities. Adverse events will be monitored using the NCI CTCAE Version 4.0.
- Laboratory testing CBC with differential/platelets
- Serum chemistry studies to include sodium, potassium, chloride, carbon dioxide, BUN, creatinine, glucose, magnesium, calcium, phosphorus, albumin, total protein, SGOT (AST) or SGPT (AGT), total bilirubin, alkaline phosphatase, cholesterol, triglycerides and uric acid.
- Routine urinalysis
- Optional serum biomarkers (expansion cohort)
- Optional biopsy (expansion cohort, GIST patients only)

7.2.3. *At the end of every 2$^{nd}$ study cycle and every 2-cycles after the last dose of ipilimumab is administered*

- Physical examination to include height, weight and vital signs.
- Physical examination, interim history pertaining to any change from baseline, current medications and treatment-related toxicities. Adverse events will be monitored using the NCI CTCAE Version 4.0.
- Laboratory testing CBC with differential/platelets

- Serum chemistry studies to include sodium, potassium, chloride, carbon dioxide, BUN, creatinine, glucose, magnesium, calcium, phosphorus, albumin, total protein, SGOT (AST) or SGPT (AGT), total bilirubin, alkaline phosphatase, cholesterol, triglycerides and uric acid.
- Routine urinalysis
- ECG
- Imaging studies: Re-staging scans or other disease assessment diagnostics to determine response:
  - o Contrast CT scans of the abdomen and pelvis (preferred) or MRI of abdomen and pelvis
  - o Chest X-ray (if lung metastases are evident on chest x-ray, CT of the chest should be obtained as well)
  - o Bone scan
- Serum biomarkers (collected under protocol PA13-0291)

**7.3. Measurement of Effect**

Patients with measurable disease will be assessed by standard criteria.

1. Definitions:

Response and progression will be evaluated in this study using guidelines proposed by the Immune Related Response Criteria (irRC) and Choi criteria (GIST tumors only).

**irRC: Measurable Disease**

Index lesions: Must be accurately measured in two dimensions, with a minimum size of ≥5 x 5mm (two largest perpendicular diameters) by CT scan (CT scan slice thickness no greater than 5 mm) or 10mm caliper measurement by clinical exam (lesions which cannot be accurately measured with calipers should be recorded as non-measurable).

**irRC: Index and non-Index Lesions:**

For the irRC, only index and measurable new lesions are taken into account (in contrast to conventional WHO criteria, which do not require the measurement of new lesions, nor do they include new lesion measurements in the characterization of evolving tumor burden). At the baseline tumor assessment, the sum of the products of the two largest perpendicular diameters (SPD) of all index lesions (five lesions per organ, up to 10 visceral lesions and five cutaneous index lesions) is calculated. At each subsequent tumor assessment, the SPD of the index lesions and of new, measurable lesions (≥5 x 5 mm; up to 5 new lesions per organ: 5 new cutaneous lesions and 10 visceral lesions) are added together to provide the total tumor burden.

**7.4. Guidelines for Evaluation of Measurable Disease**

All measurements should be taken and recorded in metric notation using a ruler or calipers. All baseline evaluations should be performed as closely as possible to the beginning of treatment and never more than 4 weeks before the beginning of the treatment. The same method of assessment and the same technique should be used to characterize each

identified and reported lesion at baseline and during follow-up. Imaging-based evaluation is preferred to evaluation by clinical examination when both methods have been used to assess the antitumor effect of a treatment.

**Clinical lesions**. Clinical lesions will only be considered measurable when they are superficial (e.g., skin nodules and palpable lymph nodes). In the case of skin lesions, documentation by color photography, including a ruler to estimate the size of the lesion, is recommended.

**Chest x-ray.** Lesions on chest X-ray are acceptable as measurable lesions when they are clearly defined and surrounded by aerated lung. However, CT is preferable.

Helical (or Spiral) CT and MRI. These techniques should be performed with cuts of 10 mm or less in slice thickness contiguously. Spiral CT should be performed using a ≤5 mm contiguous reconstruction algorithm. This applies to tumors of the chest, abdomen, and pelvis. Head and neck tumors and those of extremities usually require specific protocols. Ultrasound (US). When the primary endpoint of the study is objective response evaluation, US should not be used to measure tumor lesions. It is, however, a possible alternative to clinical measurements of superficial palpable lymph nodes, subcutaneous lesions, and thyroid nodules. US might also be useful to confirm the complete disappearance of superficial lesions usually assessed by clinical examination.

**Endoscopy, Laparoscopy**. These will not be used to assess response on this study.

Tumor markers. Tumor markers alone cannot be used to assess response. If markers are initially above the upper normal limit, they must normalize for a patient to be considered in complete clinical response.

**Cytology, Histology**. These techniques can be used to differentiate between partial responses (PR) and complete responses (CR) if clinically indicated.

## 7.5. Response Criteria

7.5.1. *Immune Related Response Criteria (irRC)*

**Evaluation of Target Lesions**

Response in new patients will be conducted using the Immune Related Response Criteria (irRC), as described by Wolchok, et al 60. “For irRC, only index and measurable new lesions are taken into account. At the baseline tumor assessment, the sum of the products of the two largest perpendicular diameters (SPD) of all index lesions (five lesions per organ, up to 10 visceral lesions and five cutaneous index lesions) is calculated. At each subsequent tumor assessment, the SPD of the index lesions and of new, measurable lesions (≥5 x 5mm; up to 5 new lesions per organ: 5 new cutaneous lesions and 10 visceral lesions) are added together to provide the total tumor burden:”

Tumor Burden=SPDindex lesions + SPDnew, measurable lesions

**Complete Response (irCR):** irCR, complete disappearance of all lesions (whether measurable or not, and no new lesions) confirmation by a repeat, consecutive assessment no less than 4 wk from the date first documented.

**Partial Response (irPR):** irPR, decrease in tumor burden ≥50% relative to baseline confirmed by a consecutive assessment at least 4 wk after first documentation.

**Progressive Disease (irPD):** irPD, increase in tumor burden ≥25% relative to nadir (minimum recorded tumor burden) confirmation by a repeat, consecutive assessment no less than 4 wk from the date first documented.

2. If a patient is classified as having irPD at a post-baseline tumor assessment, then confirmation of irPD by a second scan in the absence of rapid clinical deterioration is required. The definition of confirmation of progression represents an increase in tumor burden ≥25% compared with the nadir at two consecutive time points at least 4 wk apart. It is recommended that this be done at the discretion of the investigator because follow-up with observation alone may not be appropriate for patients with a rapid decline in performance status.

**Stable Disease (irSD):** irSD, not meeting criteria for irCR or irPR, in absence of irPD.

**Table 2.** Derivation of irRC overall responses

| Measurable response | Nonmeasurable response | | Overall response |
|---|---|---|---|
| Index and new, measurable lesions (tumor burden),* % | Non-index lesions | New, nonmeasurable lesions | Using irRC |
| ↓100 | Absent | Absent | irCR† |
| ↓100 | Stable | Any | irPR† |
| ↓100 | Unequivocal progression | Any | irPR† |
| ↓≥50 | Absent/Stable | Any | irPR† |
| ↓≥50 | Unequivocal progression | Any | irPR† |
| ↓<50 to <25↑ | Absent/Stable | Any | irSD |
| ↓<50 to <25↑ | Unequivocal progression | Any | irSD |
| ≥25? | Any | Any | irPD† |

*Decreases assessed relative to baseline, including measurable lesions only (>5 × 5 mm).
†Assuming response (irCR) and progression (irPD) are confirmed by a second, consecutive assessment at least 4 wk apart.

7.5.2. *Choi Response Criteria for GIST*

Choi response criteria will be used in addition to irRC for assessing tumor response in GIST patients. Tumor response by this criteria is defined as a 10% decrease in the unidimensional tumor size or a 15% decrease in tumor density, as measured in Hounsfield units (HU) (45).

**Table 1.** Comparison between WHO criteria and the irRC

| | WHO | irRC |
|---|---|---|
| New, measurable lesions (i.e., ≥5 × 5 mm) | Always represent PD | Incorporated into tumor burden |
| New, nonmeasurable lesions (i.e., <5 × 5 mm) | Always represent PD | Do not define progression (but preclude irCR) |
| Non-index lesions | Changes contribute to defining BOR of CR, PR, SD, and PD | Contribute to defining irCR (complete disappearance required) |
| CR | Disappearance of all lesions in two consecutive observations not less than 4 wk apart | Disappearance of all lesions in two consecutive observations not less than 4 wk apart |
| PR | ≥50% decrease in SPD of all index lesions compared with baseline in two observations at least 4 wk apart, in absence of new lesions or unequivocal progression of non-index lesions | ≥50% decrease in tumor burden compared with baseline in two observations at least 4 wk apart |
| SD | 50% decrease in SPD compared with baseline cannot be established nor 25% increase compared with nadir, in absence of new lesions or unequivocal progression of non-index lesions | 50% decrease in tumor burden compared with baseline cannot be established nor 25% increase compared with nadir |
| PD | At least 25% increase in SPD compared with nadir and/or unequivocal progression of non-index lesions and/or appearance of new lesions (at any single time point) | At least 25% increase in tumor burden compared with nadir (at any single time point) in two consecutive observations at least 4 wk apart |

### 7.6. Biomarkers

Tumor-specific antigens that can elicit cellular and humoral immunity, are expressed on cancer cells and can be identified for development of immunotherapy in these patients. The objective of this study is to identify tumor-associated antigens or genes that elicit cellular and humoral immune responses in patients with solid tumors. This analysis will correlate antigen-expression and immune responses with patient data such as tumor type, treatment response, and clinical outcome of patients who have received ipilimumab and imatinib mesylate combination therapy.

This study will be done in collaboration with Dr. Padmanee Sharma, MD/PhD, and Dr. James Allison, PhD and covered by the immunotherapy platform supported by MD Anderson Laboratory Protocol PA13-0291. All samples will be collected using procedures characterized in this protocol and all patients will be consented for procedures done under this protocol separately. Specifically, this study will allow for the collection of upto cc of blood, to be drawn at the time of routine blood-draw,  to be used for biomarker analysis. Serum samples will be collected in 10 cc green top, heparin tubes for biomarker analysis. Biopsies will be collected before drug initiation and two weeks (± 1 week) after drug administration in *KIT* positive GIST (n= 10-15) patients of the expansion cohort only, and patients will be consented under protocol PA13-0291 for the tumor biopsies.

## 8. Adverse Event Reporting

### 8.1. Serious Adverse Event Reporting (SAE)

A serious adverse event is – any adverse drug experience occurring at any dose that results in any of the following outcomes:

3. Death
4. A life-threatening adverse drug experience – any adverse experience that places the patient, in the view of the initial reporter, at immediate risk of death from the adverse experience as it occurred. It does not include an adverse experience that, had it occurred in a more severe form, might have caused death.

5. Inpatient hospitalization or prolongation of existing hospitalization*
6. A persistent or significant disability/incapacity – a substantial disruption of a person's ability to conduct normal life functions.
7. A congenital anomaly/birth defect.

NOTE: Immune-mediated adverse reactions are expected and well described in the package insert for ipilimumab. Hospitalizations required for intravenous administration of high dose steroids will be considered adverse events and will not be considered SAEs. If the adverse event has not improved within 7 days of intravenous high dose steroids, it will be deemed serious and reported in an expedited manner to the appropriate groups.

Important medical events that may not result in death, be life-threatening, or require hospitalization may be considered a serious adverse drug experience when, based upon appropriate medical judgment, they may jeopardize the patient or subject and may require medical or surgical intervention to prevent one of the outcomes listed in this definition. Examples of such medical events include allergic bronchospasm requiring intensive treatment in an emergency room or at home, blood dyscrasias or convulsions that do not result in inpatient hospitalization, or the development of drug dependency or drug abuse (21 CFR 312.32).

Important medical events as defined above, may also be considered serious adverse events. Any important medical event can and should be reported as an SAE if deemed appropriate by the Principal Investigator or the IND Sponsor, Investigational New Drug (IND) Office.

All events occurring during the conduct of a protocol and meeting the definition of a SAE must be reported to the IRB in accordance with the timeframes and procedures outlined in "University of Texas M. D. Anderson Cancer Center Institutional Review Board Policy on Reporting Serious Adverse Events". Unless stated otherwise in the protocol, all SAEs, expected or unexpected, must be reported to IND Office, regardless of attribution (within 5 working days of knowledge of the event).

All life-threatening or fatal events with possible, probable or definite attribution to the study drug must have a written report faxed within 24 hours (next working day) of knowledge of the event to the Safety Project Manager in the IND Office.

The MDACC Internal Adverse Event Reporting Form will be used for reporting to the Sponsor (Safety Project Manager IND Office).

Serious adverse events will be captured from the time the patient signs consent until 30 days after the last dose of drug. Serious adverse events must be followed until clinical recovery is complete and laboratory test have returned to baseline, progression of the event has stabilized, or there has been acceptable resolution of the event.

Additionally, any serious adverse events that occur after the 30 day time period that are related to the study treatment must be reported to the IRB and the Sponsor (Safety Project Manager IND Office). This may include the development of a secondary malignancy.

### 8.2. Reporting of Adverse Events

This study uses FDA approved agents with known toxicity profiles. Therefore, Grade 1 and 2 toxicities (related or unrelated) will not be collected or documented as these are not considered clinically significant in this patient population and/or they are expected for these study agents. Grade 3 and 4 toxicities that are felt to be treatment related and unexpected (per package insert) will be documented. Unless otherwise documented in the electronic medical record as clinically significant and study drug related, all lab abnormalities will be assumed to be related to the patient's other co-morbid conditions, prior therapies, other concomitant therapies/medications, or underlying cancer. Adverse Events will be documented according to the Recommended Adverse Event Recording Guidelines for Phase I protocol.

It is the responsibility of the PI and the research teams to ensure serious adverse events are reported according to the Code of Federal Regulations, Good Clinical Practices, the protocol guidelines, the sponsor's guidelines, and Institutional Review Board policy.

## 9. Statistical Considerations

**Data Collection**

All patients who meet eligibility criteria and are enrolled in this trial will be registered in Clinical Oncology Research Database at the University of Texas M D Anderson Cancer Center at Houston.

**Data Protection and Confidentiality**

All patients who meet eligibility criteria and are enrolled in this trial will be registered in Clinical Oncology Research Database (CORe) at the University of Texas M D Anderson Cancer Center at Houston. All protocol participants must be registered in the CORE. The date in the current informed consent document is displayed to ensure only the most current IRB approved version is used. Consent date, registration date, off study date, and evaluability data are required for all registrants.

The principal investigator agrees to keep all information and results concerning the study confidential. The confidentiality obligation applies to all personnel involved with this clinical trial. The Investigator must ensure that each participant's anonymity will be maintained in accordance with applicable laws. The principal investigator should keep a separate log of ID numbers, names and addresses. Documents that contain the names associated with these ID numbers (e.g., written consent/assent forms), should be maintained by the Investigator in strict confidence except to the extent necessary to allow auditing by regulatory authorities, auditing or monitoring by the IRB.

The Principal Investigator shall obtain all such permissions and authorizations as may be necessary or desirable to allow the collection and use of information protected under federal privacy laws and state privacy laws, including permission/authorization for monitoring and analysis (including re-analysis in combination with results of other studies), for regulatory submission purposes and for applicable reporting (if any).

### 9.1. Data Set Descriptions

This study will utilize a standard 3+3 design and dose escalation will proceed according to the following scheme:

| Number of Patients with DLT at a Given Dose Level | Escalation Decision Rule |
|---|---|
| 0 out of 3 | Enter 3 patients at the next dose level. |
| ≥2 | Dose escalation will be stopped. This dose level will be declared the maximally administered dose (highest dose administered). Three (3) additional patients will be entered at the next lowest dose level if only 3 patients were treated previously at that dose. |
| 1 out of 3 | Enter at least 3 more patients at this dose level.<br><br>If 0 of these 3 patients experience DLT, proceed to the next dose level.<br><br>If 1 or more of this group suffer DLT, then dose escalation is stopped, and this dose is declared the maximally administered dose. Three (3) additional patients will be entered at the next lowest dose level if only 3 patients were treated previously at that dose. |
| **MTD:** The highest dose at which no more than 1 of 6 evaluable patients has had a DLT. Six patients should be treated before the dose is declared the MTD. | |

Additional subjects may be enrolled at or below the current dose level if the Investigator determines that additional safety, PK, and/or pharmacodynamic data should be obtained in order to help determine the safety and/or biologic activity at any given dose level. Additional subjects will generally be accrued in multiples of three, or until DLT is observed in ≥ 33% of subjects treated. This may be done concurrent with the higher dose cohort.

If by Dose Level 4 the MTD is not defined, then the MTD will be Dose Level 4.

Once the MTD or MTD is determined, an additional 20 patients will be enrolled in each cohort (c-kit mutated GIST, melanoma, any solid tumors) at this dose to further define this dose and help determine biological endpoints. Should DLT occur in more than 33% of

patients enrolled into this expanded cohort, dosing at that level will be stopped. The next lower dose level studied will be considered the MTD. A review of all DLTs observed during the expansion cohort will be conducted, and the need to lower the dose will be based on the discussion and agreement between the IRB and the Investigator.

All patients who receive any study drug will be considered evaluable for response and will be included in the efficacy data set.

9.1.1. Safety Evaluation

The incidence of clinical and laboratory adverse events will be reported and graded according to the NCI-CTCAE version 4.0 (available at http://ctep.cancer.gov/reporting/ctc.html). Adverse events will be reported in frequency tables overall, by intensity, and by relationship. Laboratory values will be reported in shift tables and with summary statistics.

9.1.2. Efficacy Evaluation

Preliminary information on the anti-tumor activity of ipilimumab and imatinib combination therapy will be collected in arm 2 of this study. Tumor response will be determined using the Immune Related Response Criteria (irRC). Efficacy evaluations using radiologic assessments will be performed every 2 cycles of study treatment. The same radiologic procedures used to define measurable or non-measurable disease at baseline must be used throughout the study.

**9.2. Analysis**

9.2.1. Statistical Analysis
Descriptive statistics will be computed for all relevant outcomes, including tumor response, and biomarker response (as described in 2005-0027).

9.2.2. Demographics and Baseline Characteristics
Frequency distributions of gender and race will be tabulated by dose/cohort. Summary statistics for age, body weight, height, and Body Mass Index (BMI) will be tabulated by dose/cohort.

9.2.3. Safety Analyses
All recorded adverse events will be recorded per MDACC guidelines using CTCAE Version 4.0.

9.2.4. Efficacy Analyses
Tables and listings will be provided for the efficacy measurements.

9.2.5. Sample Size/Accrual Rate
The maximum planned number of patients will be 96 (9 per dose level plus 20 patients per cohort in the expansion study). It is estimated that approximately 3 patients per month will be accrued and a four week interval will be allowed between dose levels.

9.2.6. Stratification Factors
This study will not utilize stratification factors.

## 10. References

1. Tarhini AA, Iqbal F. CTLA-4 blockade: therapeutic potential in cancer treatments. Onco Targets Ther2010;3:15-25.

2. Demetri GD, von Mehren M, Blanke CD, Van den Abbeele AD, Eisenberg B, Roberts PJ, Heinrich MC, Tuveson DA, Singer S, Janicek M, Fletcher JA, Silverman SG, Silberman SL, Capdeville R, Kiese B, Peng B, Dimitrijevic S, Druker BJ, Corless C, Fletcher CD, Joensuu H. Efficacy and safety of imatinib mesylate in advanced gastrointestinal stromal tumors. N Engl J Med2002 Aug 15;347(7):472-80.

3. Blanke CD, Rankin C, Demetri GD, Ryan CW, von Mehren M, Benjamin RS, Raymond AK, Bramwell VH, Baker LH, Maki RG, Tanaka M, Hecht JR, Heinrich MC, Fletcher CD, Crowley JJ, Borden EC. Phase III randomized, intergroup trial assessing imatinib mesylate at two dose levels in patients with unresectable or metastatic gastrointestinal stromal tumors expressing the kit receptor tyrosine kinase: S0033. J Clin Oncol2008 Feb 1;26(4):626-32.

4. Balachandran VP, Cavnar MJ, Zeng S, Bamboat ZM, Ocuin LM, Obaid H, Sorenson EC, Popow R, Ariyan C, Rossi F, Besmer P, Guo T, Antonescu CR, Taguchi T, Yuan J, Wolchok JD, Allison JP, DeMatteo RP. Imatinib potentiates antitumor T cell responses in gastrointestinal stromal tumor through the inhibition of Ido. Nature medicine2011 Sep;17(9):1094-100.

5. Weber J. Review: anti-CTLA-4 antibody ipilimumab: case studies of clinical response and immune-related adverse events. Oncologist2007 Jul;12(7):864-72.

6. Hodi FS, O'Day SJ, McDermott DF, Weber RW, Sosman JA, Haanen JB, Gonzalez R, Robert C, Schadendorf D, Hassel JC, Akerley W, van den Eertwegh AJ, Lutzky J, Lorigan P, Vaubel JM, Linette GP, Hogg D, Ottensmeier CH, Lebbe C, Peschel C, Quirt I, Clark JI, Wolchok JD, Weber JS, Tian J, Yellin MJ, Nichol GM, Hoos A, Urba WJ. Improved survival with ipilimumab in patients with metastatic melanoma. N Engl J Med2010 Aug 19;363(8):711-23.

7. Robert C, Thomas L, Bondarenko I, O'Day S, M DJ, Garbe C, Lebbe C, Baurain JF, Testori A, Grob JJ, Davidson N, Richards J, Maio M, Hauschild A, Miller WH, Jr., Gascon P, Lotem M, Harmankaya K, Ibrahim R, Francis S, Chen TT, Humphrey R, Hoos A, Wolchok JD. Ipilimumab plus dacarbazine for previously untreated metastatic melanoma. N Engl J Med2011 Jun 30;364(26):2517-26.

8. Druker BJ, Tamura S, Buchdunger E, Ohno S, Segal GM, Fanning S, Zimmermann J, Lydon NB. Effects of a selective inhibitor of the Abl tyrosine kinase on the growth of Bcr-Abl positive cells. Nature medicine1996 May;2(5):561-6.

9. Buchdunger E, Cioffi CL, Law N, Stover D, Ohno-Jones S, Druker BJ, Lydon NB. Abl protein-tyrosine kinase inhibitor STI571 inhibits in vitro signal transduction mediated by c-kit and platelet-derived growth factor receptors. The Journal of pharmacology and experimental therapeutics2000 Oct;295(1):139-45.

10. Carroll M, Ohno-Jones S, Tamura S, Buchdunger E, Zimmermann J, Lydon NB, Gilliland DG, Druker BJ. CGP 57148, a tyrosine kinase inhibitor, inhibits the growth of cells expressing BCR-ABL, TEL-ABL, and TEL-PDGFR fusion proteins. Blood1997 Dec 15;90(12):4947-52.

11. Buchdunger E, Zimmermann J, Mett H, Meyer T, Muller M, Druker BJ, Lydon NB. Inhibition of the Abl protein-tyrosine kinase in vitro and in vivo by a 2-phenylaminopyrimidine derivative. Cancer Res1996 Jan 1;56(1):100-4.

12. Druker BJ, Talpaz M, Resta DJ, Peng B, Buchdunger E, Ford JM, Lydon NB, Kantarjian H, Capdeville R, Ohno-Jones S, Sawyers CL. Efficacy and safety of a specific inhibitor of the BCR-ABL tyrosine kinase in chronic myeloid leukemia. N Engl J Med2001 Apr 5;344(14):1031-7.
13. van Oosterom AT, Judson IR, Verweij J, Stroobants S, Dumez H, Donato di Paola E, Sciot R, Van Glabbeke M, Dimitrijevic S, Nielsen OS. Update of phase I study of imatinib (STI571) in advanced soft tissue sarcomas and gastrointestinal stromal tumors: a report of the EORTC Soft Tissue and Bone Sarcoma Group. Eur J Cancer2002 Sep;38 Suppl 5:S83-7.
14. van Oosterom AT, Judson I, Verweij J, Stroobants S, Donato di Paola E, Dimitrijevic S, Martens M, Webb A, Sciot R, Van Glabbeke M, Silberman S, Nielsen OS. Safety and efficacy of imatinib (STI571) in metastatic gastrointestinal stromal tumours: a phase I study. Lancet2001 Oct 27;358(9291):1421-3.
15. Rubin BP, Heinrich MC, Corless CL. Gastrointestinal stromal tumour. Lancet2007 May 19;369(9574):1731-41.
16. Hirota S, Isozaki K, Moriyama Y, Hashimoto K, Nishida T, Ishiguro S, Kawano K, Hanada M, Kurata A, Takeda M, Muhammad Tunio G, Matsuzawa Y, Kanakura Y, Shinomura Y, Kitamura Y. Gain-of-function mutations of c-kit in human gastrointestinal stromal tumors. Science (New York, NY1998 Jan 23;279(5350):577-80.
17. Kemmer K, Corless CL, Fletcher JA, McGreevey L, Haley A, Griffith D, Cummings OW, Wait C, Town A, Heinrich MC. KIT mutations are common in testicular seminomas. Am J Pathol2004 Jan;164(1):305-13.
18. Curtin JA, Busam K, Pinkel D, Bastian BC. Somatic activation of KIT in distinct subtypes of melanoma. J Clin Oncol2006 Sep 10;24(26):4340-6.
19. Smalley KS, Nathanson KL, Flaherty KT. Genetic subgrouping of melanoma reveals new opportunities for targeted therapy. Cancer Res2009 Apr 15;69(8):3241-4.
20. Paschka P, Marcucci G, Ruppert AS, Mrozek K, Chen H, Kittles RA, Vukosavljevic T, Perrotti D, Vardiman JW, Carroll AJ, Kolitz JE, Larson RA, Bloomfield CD. Adverse prognostic significance of KIT mutations in adult acute myeloid leukemia with inv(16) and t(8;21): a Cancer and Leukemia Group B Study. J Clin Oncol2006 Aug 20;24(24):3904-11.
21. Worobec AS, Semere T, Nagata H, Metcalfe DD. Clinical correlates of the presence of the Asp816Val c-kit mutation in the peripheral blood mononuclear cells of patients with mastocytosis. Cancer1998 Nov 15;83(10):2120-9.
22. Corless CL, Fletcher JA, Heinrich MC. Biology of gastrointestinal stromal tumors. J Clin Oncol2004 Sep 15;22(18):3813-25.
23. Clary BM, DeMatteo RP, Lewis JJ, Leung D, Brennan MF. Gastrointestinal stromal tumors and leiomyosarcoma of the abdomen and retroperitoneum: a clinical comparison. Ann Surg Oncol2001 May;8(4):290-9.
24. Rubin JL, Sanon M, Taylor DC, Coombs J, Bollu V, Sirulnik L. Epidemiology, survival, and costs of localized gastrointestinal stromal tumors. Int J Gen Med2011;4:121-30.
25. Vanneman M, Dranoff G. Combining immunotherapy and targeted therapies in cancer treatment. Nat Rev Cancer2012 Apr;12(4):237-51.
26. Peng B, Dutreix C, Mehring G, Hayes MJ, Ben-Am M, Seiberling M, Pokorny R, Capdeville R, Lloyd P. Absolute bioavailability of imatinib (Glivec) orally versus intravenous infusion. J Clin Pharmacol2004 Feb;44(2):158-62.
27. le Coutre P, Kreuzer KA, Pursche S, Bonin M, Leopold T, Baskaynak G, Dorken B, Ehninger G, Ottmann O, Jenke A, Bornhauser M, Schleyer E. Pharmacokinetics and

cellular uptake of imatinib and its main metabolite CGP74588. Cancer Chemother Pharmacol2004 Apr;53(4):313-23.
28. Druker BJ. Imatinib mesylate in the treatment of chronic myeloid leukaemia. Expert Opin Pharmacother2003 Jun;4(6):963-71.
29. O'Brien SG, Meinhardt P, Bond E, Beck J, Peng B, Dutreix C, Mehring G, Milosavljev S, Huber C, Capdeville R, Fischer T. Effects of imatinib mesylate (STI571, Glivec) on the pharmacokinetics of simvastatin, a cytochrome p450 3A4 substrate, in patients with chronic myeloid leukaemia. Br J Cancer2003 Nov 17;89(10):1855-9.
30. Frye RF, Fitzgerald SM, Lagattuta TF, Hruska MW, Egorin MJ. Effect of St John's wort on imatinib mesylate pharmacokinetics. Clin Pharmacol Ther2004 Oct;76(4):323-9.
31. Avramis IA, Laug WE, Sausville EA, Avramis VI. Determination of drug synergism between the tyrosine kinase inhibitors NSC 680410 (adaphostin) and/or STI571 (imatinib mesylate, Gleevec) with cytotoxic drugs against human leukemia cell lines. Cancer Chemother Pharmacol2003 Oct;52(4):307-18.
32. Van Glabbeke M, Verweij J, Casali PG, Simes J, Le Cesne A, Reichardt P, Issels R, Judson IR, van Oosterom AT, Blay JY. Predicting toxicities for patients with advanced gastrointestinal stromal tumours treated with imatinib: a study of the European Organisation for Research and Treatment of Cancer, the Italian Sarcoma Group, and the Australasian Gastro-Intestinal Trials Group (EORTC-ISG-AGITG). Eur J Cancer2006 Sep;42(14):2277-85.
33. Joensuu H, Roberts PJ, Sarlomo-Rikala M, Andersson LC, Tervahartiala P, Tuveson D, Silberman S, Capdeville R, Dimitrijevic S, Druker B, Demetri GD. Effect of the tyrosine kinase inhibitor STI571 in a patient with a metastatic gastrointestinal stromal tumor. N Engl J Med2001 Apr 5;344(14):1052-6.
34. Verweij J, Casali PG, Zalcberg J, LeCesne A, Reichardt P, Blay JY, Issels R, van Oosterom A, Hogendoorn PC, Van Glabbeke M, Bertulli R, Judson I. Progression-free survival in gastrointestinal stromal tumours with high-dose imatinib: _andomized trial. Lancet2004 Sep 25-Oct 1;364(9440):1127-34.
35. Preventing Occupational Exposures to Antineoplastic and Other Hazardous Drugs in Health Care Settings. 2004. p. 165.
36. Manual OT. Controlling Occupational Exposure to Hazardous Drugs. In: OSHA, editor.1999.
37. Chaffee BW, Armitstead JA, Benjamin BE, Cotugno MC, Forrey RA, Hintzen BL, Pfeiffenberger T, Stevenson JG. Guidelines for the safe handling of hazardous drugs: consensus recommendations. Am J Health Syst Pharm2010 Sep 15;67(18):1545-6.
38. Jacobson JO, Polovich M, Gilmore TR, Schulmeister L, Esper P, Lefebvre KB, Neuss MN. Revisions to the 2009 american society of clinical oncology/oncology nursing society chemotherapy administration safety standards: expanding the scope to include inpatient settings. J Oncol Pract2012 Jan;8(1):2-6.
39. Weber JS, O'Day S, Urba W, Powderly J, Nichol G, Yellin M, Snively J, Hersh E. Phase I/II study of ipilimumab for patients with metastatic melanoma. J Clin Oncol2008 Dec 20;26(36):5950-6.

# 11. Study Calendars

## 11.1. Dose Escalation Study Calendar

| Assessment Tool | Baseline[3] | Cycle 0 | | Cycle 1 | | | Cycle 2 | | | Cycle 3 | | | Cycle 4 | | | Maintenance Period |
|---|---|---|---|---|---|---|---|---|---|---|---|---|---|---|---|---|
| | | Week | | | | | Week | | | Week | | | Week | | | Months |
| | | 1 | 2 | 3 | 4[7] | 5 | 1 | 2 | 3 | 1 | 2 | 3 | 1 | 2 | 3 | 1-6[9] |
| **Range for study related visits** | ± 2 days | * | * | * | * | * | * | * | * | * | * | * | * | * | * | * |
| **Imatinib mesylate** | | X[4] | X | X | X | X | X | X | X | X | X | X | X | X | X | |
| **Ipilimumab** | | | | X[6] | | | X | | | X | | | X | | | |
| **History & Physical Exam[1]** | X | | | X | | | X | | | X | | | X | | | X |
| **CBC & differential[1]** | X | | | | X | | | | X | | | | | | X | X |
| **ECG[1]** | X[8] | | | | | | | | X | | | | | | X | X |
| **Sodium, potassium, chloride, bicarbonate, BUN, creatinine, glucose, calcium, magnesium Albumin, alkaline phosphatase, total bilirubin, SGOT (AST), SGPT[ALT], cholesterol, triglycerides[1]** | X | | | | X | | | | X | | | | | | X | X |
| **Urinalysis[1]** | X | | | | X | | | | X | | | | | | X | X |
| **Serum pregnancy test (women of childbearing potential) [1]** | X | | | | X | | | | X | | | | X | | | X |
| **irRC imaging and tumor assessment** | X[2] | | | | | | | | X | | | | | | X | X |
| **Biomarker studies (optional)** | X[5] | | | X[5] | | | | | X[5] | | | | | | X[5] | X[5] |

* All study related visits will be allowed a range of ± 2 days.

1. Physical exams and lab tests measurements will be given a window of ± 2 days. Every patient needs to be evaluated by the physician within 7 days before start of study and ± 2 days before each subsequent ipilimumab administration. Pregnancy tests should be recorded every month.
2. Initial tumor assessment should occur within 4-weeks before start of study.
3. Baseline visit within 7 days prior to initiation of therapy.
4. Imatinib mesylate treatment will be given 14 days (± 2) prior to first ipilimumab treatment.
5. Performed under protocol PA13-0291, Serum biomarker measurements will be taken at baseline (7 days prior to therapy initiation), immediately before first ipilimumab administration, and every two cycles following first ipilimumab administration. A window of ± 2 days will be allowed.
6. First ipilimumab treatment will begin on day 15 (± 2) and start a 21 day/cycle combination therapy.
7. 1 week (± 2 days) after initiation of ipilimumab therapy (week 4) patients will be assessed for toxicity, physical exampination, CBC, serum chemistry,,urinalysis and pregnancy test.
8. A baseline ECG will be recorded within 2-weeks (± 3 days) prior to therapy initiation and.
9. Daily imatinib mesylate will be continued after completion of cycle 4. Patients will obtain hematological chemistries, physical examination, and once a month. Rstaging will occur every 2 cycles (± 1 week). If patient has SD and benefited from the trial ≥ 1 year it is at the discretion of the physician as to how frequently f/u will be required, but patients must have a minimum restaging of every 4 cycles.

### 11.2. Ipilimumab and Imatinib Mesylate MTD Combination Therapy (Expansion Cohort) Study Calendar

| Assessment Tool | Baseline[5] | Cycle 1 | | | Cycle 2 | | | Cycle 3 | | | Cycle 4 | | | Maintenance Period |
|---|---|---|---|---|---|---|---|---|---|---|---|---|---|---|
| | | Week | | | Week | | | Week | | | Week | | | Months |
| | | 1 | 2 | 3 | 1 | 2 | 3 | 1 | 2 | 3 | 1 | 2 | 3 | 1-6[8] |
| Range for study related visits | ± 2 days | * | * | * | * | * | * | * | * | * | * | * | * | * |
| Imatinib mesylate | | X[7] | X | X | X | X | X | X | X | X | X | X | X | |
| Ipilimumab | | X[7] | | | X | | | X | | | X | | | |
| History & Physical Exam[1] | X | | | | X | | | X | | | X | | | X |
| CBC & differential[1] | X | | | | | | X | | | | | | X | X |
| ECG[2] | X[2] | | | | | | X | | | | | | X | X |
| Sodium, potassium, chloride, bicarbonate, BUN, creatinine, glucose, calcium, magnesium Albumin, alkaline phosphatase, total bilirubin, SGOT (AST), SGPT[ALT], cholesterol, triglycerides[1] | X | | | | | | X | | | | | | X | X |
| Urinalysis[1] | X | | | | | | X | | | | | | X | X |
| Serum pregnancy test (women of childbearing potential) [1] | X | | | | X | | | | X | | | | X | X |
| irRC imaging and tumor assessment | X[3] | | | | | | X | | | | | | X | X |
| Biomarker studies [6] | X | | X | | | | X | | | | | | X | X |
| Biopsies (optional) | X[4] | | X[4] | | | | | | | | | | | |

* All study related visits will be allowed a range of ± 2 days.

1. Physical exams and lab tests measurements will be given a window of ± 2 days. Every patient needs to be evaluated by the physician within 7 days before start of study and ± 2 days before each subsequent ipilimumab administration.. CBC, serum chemistry, and urinalysis will be recorded every two cycles. Pregnancy tests should be recorded every month.
2. ECG will be obtained 2-weeks (± 3 days) prior to study initiation.
3. Initial tumor assessment should occur within 4-weeks before start of study.
4. Optional biopsy baseline measurements should occur within 2-weeks before start of study and at week two of first treatment cycle (± 1 week).
5. Baseline visit within 7 days prior to initiation of therapy.
6. Performed under protocol PA13-0291, Serum biomarker measurements will be taken at baseline (7 days prior to therapy initiation), at week two of first treatment cycle (± 1 week), and every two cycles following first ipilimumab administration. A window of ± 2 days will be allowed.
7. Ipilimumab and imatinib mesylate combination therapy will begin at the MTD defined by dose escalation study on day 1 with no imatinib mesylate run in. This will begin 21 day cycle 1.
8. Daily imatinib mesylate will be continued after completion of cycle 4. Patients will obtain hematological chemistries, physical examination, and once a month. Restaging will occur every 2 cycles (± 1 week). If patient has SD and benefited from the trial ≥ 1 year it is at the discretion of the physician as to how frequently f/u will be required, but patients must have a minimum restaging of every 4 cycles.

# 10. Appendix

## 10.1. Contraindicated Drugs

- Warfarin: low-molecular weight or standard heparin should be taken instead for anticoagulation therapy
- Caution is recommended when administering Gleevec with strong CYP3A4 inhibitors:

  Ketoconazole
  Itraconazole
  Clarithromycin
  Atazanavir
  Ndinavir
  Nefazodone
  Nelfinavir
  Ritonavir
  Saquinavir
  Telithromycin
  Voriconazole
  Grapefruit Juice

- Particular caution is recommended when administering Gleevec with CYP3A4 substrates that have a narrow therapeutic window:

  Alfentanil
  Cyclosporine
  Diergotamine
  Ergotamine
  Fentanyl
  Pimozide
  Quinidine
  Sirolimus
  Tacrolimus

- Gleevec can increase systemic exposure to Acetominophen and therefore caution recommended.
