# Supplementary material for: Phase I clinical trial of combination imatinib and ipilimumab in patients with advanced malignancies
Source: J Immunother Cancer. 2017 Apr 18;5:35. doi: 10.1186/s40425-017-0238-1 (PMC5394629; doi:10.1186/s40425-017-0238-1)
Supplement: Supplementary file 2 — Mutations in patients with available profiling (n = 30). Description of data: A summary of mutations identified in all patients on trial based on available profiling studies. (PDF 19 kb) [file 40425_2017_238_MOESM2_ESM.pdf]

Mutation	# of patients	Tumor types
**APC (poss Germline)**	1	Melanoma (vaginal)
**ARFRP1 amp**	1	Melanoma (cutaneous)
**ATRX**	1	Melanoma (cutaneous)
**BCL2 H20Q**	1	Mesothelioma (peritoneal)
**CDKN2A/B**	1	NSCLC (adenoca)
**EGFR K500**	1	NSCLC (adenoca)
**ERBB2**	1	Salivary duct
**EWSR1-ATF1 fusion**	1	Melanoma (cutaneous)
**FBXW7 R465C**	1	Anal cancer
**GNA11**	1	Melanoma (uveal)
**IDH1**	1	GIST
**IGF1R**	1	GIST
**KDR**	2	Salivary duct, RCC
**KIT**	17	Melanoma (cutaneous, uveal, vaginal, vulvar), NSCLC (adenoca), RCC, Salivary duct, GIST
**KRAS G12V**	1	NSCLC (adenoca)
**MET**	2	Melanoma (vulvar), NSCLC (squamous)
**MYC**	1	GIST
**NOTCH**	1	Melanoma (cutaneous)
**NRAS**	4	Melanoma (cutaneous), GIST
**PBRM1**	1	RCC
**PDGFRA**	1	Salivary duct
**PIK3CA**	2	RCC, GIST
**PTEN**	1	Melanoma (cutaneous)
**SETD2**	1	RCC
**SMAD4**	1	RCC
**SMARCB1 loss**	1	Renal medullary
**STK11**	1	NSCLC (adenoca)
**TP53**	3	Melanoma (cutaneous), Salivary duct, Renal medullary
**VHL R210fs*6+**	1	RCC
