# Supplementary material for: Phase I clinical trial of combination imatinib and ipilimumab in patients with advanced malignancies
Source: J Immunother Cancer. 2017 Apr 18;5:35. doi: 10.1186/s40425-017-0238-1 (PMC5394629; doi:10.1186/s40425-017-0238-1)
Supplement: Supplementary file 3 — Flow characteristics of patient PBMCs during the course of treatment. Description of data: The raw percentages of CD3+ cells with expression of various surface markers expressed on CD4+ or CD8+ T-cells. (PDF 63 kb) [file 40425_2017_238_MOESM3_ESM.pdf]

Patient Acct #	Cell population	Baseline	Post imatinib/ Pre IPI	Post IPI #1	Post IPI #2	Post IPI #3
5						
	CD3+ Lymphocytes			66.2	50	56.7
	CD3+ CD4+			48	36	46.3
	CD3+ CD8+			49.2	58.8	51.1
	CD4+ 41BB+			0.48	0.48	7.63
	CD4+ CTLA4+			23.1	30.6	34.7
	CD4+ ICOS+			57.6	54.1	57.8
	CD4+ OX40+			31.4	30.7	35.4
	CD4+ PD1+			18.8	16.9	23.6
	CD8+ 41BB+			2.38	0.86	2.82
	CD8+ CTLA4+			3.29	7.85	15.1
	CD8+ ICOS+			21.2	18.4	26.9
	CD8+ OX40+			1.23	2.82	1
	CD8+ PD1+			29.4	27.8	38.9
43						
	CD3+ Lymphocytes		56	57	67.1	
	CD3+ CD4+		25.9	27	31.5	
	CD3+ CD8+		54	65.9	62.5	
	CD4+ 41BB+		7.77	7.64	6.25	
	CD4+ CTLA4+		30.1	30.6	22	
	CD4+ ICOS+		42.1	39.4	54.6	
	CD4+ OX40+		24.9	24.8	21.7	
	CD4+ PD1+		19	21	13.2	
	CD8+ 41BB+		3.29	1.97	1.32	
	CD8+ CTLA4+		21.4	18.7	9.36	
	CD8+ ICOS+		24.9	17.6	25.9	
	CD8+ OX40+		1.79	1.79	0.78	
	CD8+ PD1+		11.1	10.8	6.58	
47						
	CD3+ Lymphocytes		16.9	3.19		
	CD3+ CD4+		84.1	78.1		
	CD3+ CD8+		11.8	15.8		
	CD4+ 41BB+		8.57	12.8		
	CD4+ CTLA4+		31.2	46.6		
	CD4+ ICOS+		34.1	39.5		
	CD4+ OX40+		27.1	29.1		
	CD4+ PD1+		15.8	15.1		
	CD8+ 41BB+		3.21	7.09		
	CD8+ CTLA4+		16	29.7		
	CD8+ ICOS+		19.3	26.5		
	CD8+ OX40+		1.91	2.01		
	CD8+ PD1+		9.92	11.9		
51						
	CD3+ Lymphocytes	6.02	61.6			
	CD3+ CD4+	72.4	77			

	CD3+ CD8+	21.9	20.1			
	CD4+ 41BB+	3.34	6.9			
	CD4+ CTLA4+	13.7	28.7			
	CD4+ ICOS+	32.4	30.5			
	CD4+ OX40+	34.6	34.7			
	CD4+ PD1+	13.8	9.68			
	CD8+ 41BB+	1.5	1.98			
	CD8+ CTLA4+	3.99	13.5			
	CD8+ ICOS+	14.8	18.3			
	CD8+ OX40+	3.32	3.27			
	CD8+ PD1+	30.3	26			
66						
	CD3+ Lymphocytes	29.6	44.2	23	19.3	
	CD3+ CD4+	23.8	32.9	30.5	34.6	
	CD3+ CD8+	66.3	56.9	59	52.2	
	CD4+ 41BB+	6.51	9.73	6.1	11	
	CD4+ CTLA4+	24.5	35.4	16.4	31.3	
	CD4+ ICOS+	54	41.3	48.7	61.8	
	CD4+ OX40+	37.9	22.8	33.6	38.1	
	CD4+ PD1+	18.5	15	45.6	18.3	
	CD8+ 41BB+	1.54	11.7	3.25	3.74	
	CD8+ CTLA4+	2.73	21.2	5.06	5.32	
	CD8+ ICOS+	14.6	20.5	12.9	14.7	
	CD8+ OX40+	2.05	2.41	1.53	1.12	
	CD8+ PD1+	14	13.1	44.3	13.2	
