# Supplementary material for: Phase I clinical trial of combination imatinib and ipilimumab in patients with advanced malignancies
Source: J Immunother Cancer. 2017 Apr 18;5:35. doi: 10.1186/s40425-017-0238-1 (PMC5394629; doi:10.1186/s40425-017-0238-1)
Supplement: Supplementary file 4 — Average change from baseline among WBC count and subpopulations over the course of treatment. Description of data: A summary of average changes in cell counts over the course of treatment from individual patient baselines. (PDF 67 kb) [file 40425_2017_238_MOESM4_ESM.pdf]

Patients by response	WBC	ANC	ALC	AMC
**PD**	-0.0340909	-0.0469048	-0.248134	0.2965686
**PD**	0.1754386	0.2098139	0.0623306	0.2380952
**PD**	0.4935065	0.8004053	-0.354497	0.5
**PD**	0.121519	0.1784348	-0.165414	0.3492063
**PD**	-0.3217054	-0.3568607	-0.101553	0.0546139
**PD**	0.4019608	1.3739035	-0.14	-0.1825397
**PD**	-0.1135135	-0.0309979	-0.244444	-0.1464789
**PD**	0.3583333	0.6304094	0.0548433	0.1725146
**PD**	-0.2805195	-0.3897881	0.0331361	-0.3638889
**PD**	-0.0588235	-0.092228	-0.179012	0.5461538
**PD**	0.1848485	0.3446181	-0.170316	-0.2118056
**PD**	-0.2650273	-0.2955247	-0.202532	-0.2700422
**PD**	0.1234568	0.5235294	-0.09407	-0.1697531
**PD**	-0.2181373	-0.2589952	0.0074074	-0.2077922
**PD**	-0.1475	-0.1621622	-0.222143	-0.3191489
**PD**	-0.002381	0.0372279	-0.181818	-0.1414474
**PD**	0.0155556	0.3071274	-0.361943	-0.2088889
**group average**	**0.0254659**	**0.1630593**	**-0.147539**	**-0.0038019**
**SD**	-0.1997019	-0.2488157	-0.193798	-0.0575139
**SD**	0.1328358	0.2927649	-0.146667	0.0363636
**SD**	0.2122347	0.311719	-0.208333	0.1445783
**SD**	-0.0725309	-0.1288255	0.5183824	-0.4107143
**SD**	-0.0261905	-0.1228845	0.1331361	0.0055556
**group average**	**0.0093294**	**0.0207916**	**0.020544**	**-0.0563461**
**PR**	-0.328125	-0.3746537	-0.174296	-0.2906977
**PR**	0.0360169	0.1491337	-0.065309	-0.2208807
**group average**	**-0.146054**	**-0.11276**	**-0.119802**	**-0.2557892**
**T-test comparing changes between response groups**				
**PD vs SD**	0.4460259	0.2637977	0.0412629	0.3580622
**PD vs PR**	0.1813898	0.2183298	0.3865184	0.1275344

Supplemental table 2: Average change from baseline among WBC count and subpopulations over the course of treatment. Note: A positive number represents an increase from baseline, while a negative number represents a decrease from baseline.
