# Supplementary figures and images for: Phase I clinical trial of combination imatinib and ipilimumab in patients with advanced malignancies
Source: J Immunother Cancer. 2017 Apr 18;5:35. doi: 10.1186/s40425-017-0238-1 (PMC5394629; doi:10.1186/s40425-017-0238-1)

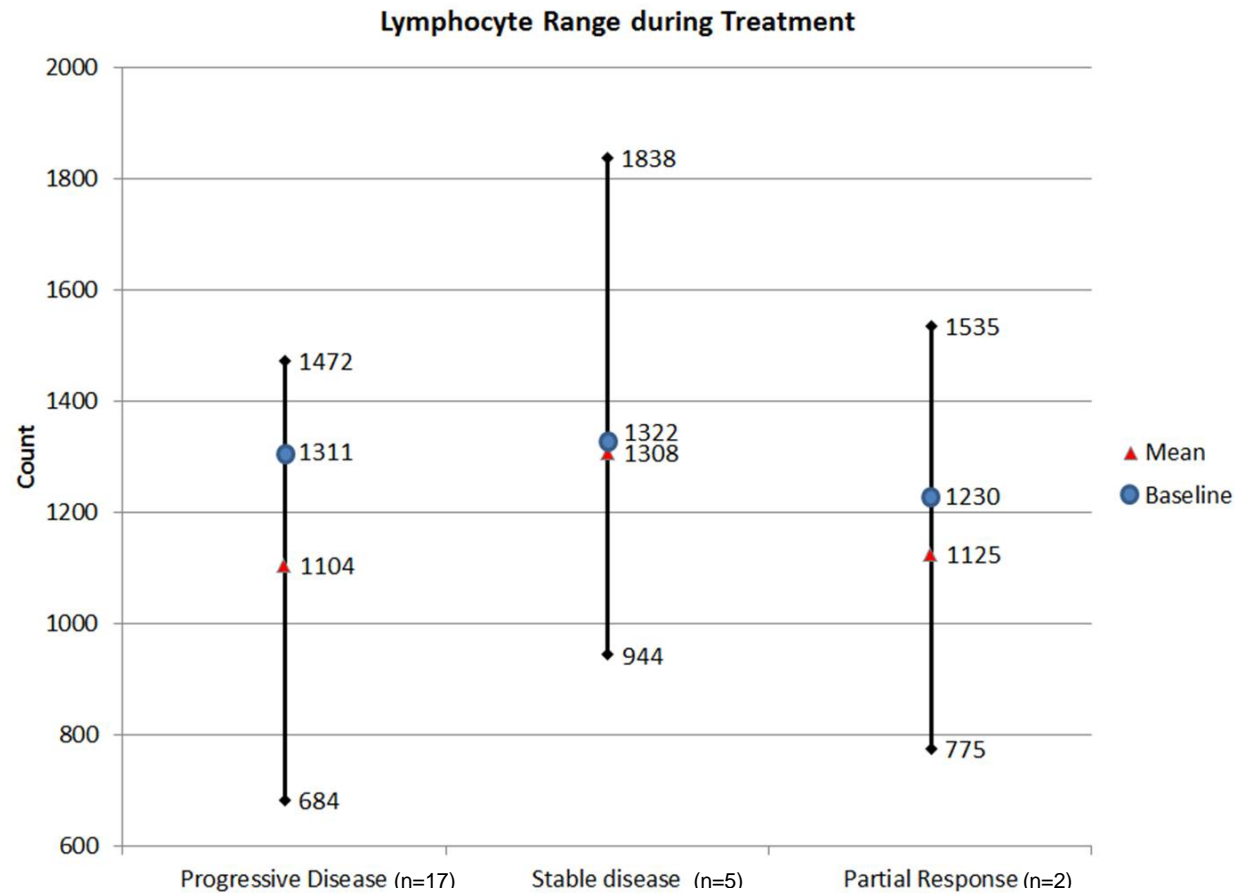

Supplemental Figure 1: Lymphocyte range during treatment.

Supplement: Supplementary file 5 — Lymphocyte range during treatment. Description of data: A comparison of average lymphocyte range over the course of treatment between patients with stable disease, progression, or response. Baseline and mean counts during treatment are shown as well as range. (PDF 76 kb) [file 40425_2017_238_MOESM5_ESM.pdf]
